# Gut bacterial consortium enriched in a biofloc system protects shrimp against *Vibrio parahaemolyticus* infection

**DOI:** 10.1186/s40168-023-01663-2

**Published:** 2023-10-19

**Authors:** Haipeng Guo, Xuezhi Fu, Jikun He, Ruoyu Wang, Mengchen Yan, Jing Wang, Pengsheng Dong, Lei Huang, Demin Zhang

**Affiliations:** 1 State Key Laboratory for Managing Biotic and Chemical Threats to the Quality and Safety of Agro-Products, Ningbo University, Ningbo, 315211 China; 2 School of Marine Sciences, Ningbo University, Ningbo, 315211 China

**Keywords:** *Penaeus vannamei*, Biofloc system, *Vibrio parahaemolyticus* resistance, Cross-transplantation, Synthetic community

## Abstract

**Background:**

Shrimp cultured in a biofloc system (BFS) have a lower disease incidence than those farmed in a water exchange system (WES). Although a number of studies have reported that the gut bacterial community induced by BFS is highly associated with shrimp disease resistance, the causal relationship remains unknown. Here, the promotive roles of gut bacterial community induced by BFS in pathogenic *Vibrio* infection resistance and its potential micro-ecological and physiological mechanisms were investigated by gut bacterial consortium transplantation and synthetic community (SynCom) construction.

**Results:**

The BFS induced a more stable and resistant gut bacterial community, and significantly enriched some beneficial bacterial taxa, such as *Paracoccus*, *Ruegeria*, *Microbacterium*, *Demequina*, and *Tenacibaculum*. Transplantation of a gut bacterial consortium from BFS shrimp (Enrich^BFS^) greatly enhanced the stability of the bacterial community and resistance against pathogenic *V. parahaemolyticus* infection in WES shrimp, while transplantation of a gut bacterial consortium from WES shrimp significantly disrupted the bacterial community and increased pathogen susceptibility in both WES and BFS shrimp. The addition of Enrich^BFS^ in shrimp postlarvae also improved the pathogen resistance through increasing the relative abundances of beneficial bacterial taxa and stability of bacterial community. The corresponding strains of five beneficial bacterial taxa enriched in BFS shrimp were isolated to construct a SynCom^BFS^. The addition of SynCom^BFS^ could not only suppress disease development, but also improve shrimp growth, boost the digestive and immune activities, and restore health in diseased shrimp. Furthermore, the strains of SynCom^BFS^ well colonized shrimp gut to maintain a high stability of bacterial community.

**Conclusions:**

Our study reveals an important role for native microbiota in protecting shrimp from bacterial pathogens and provides a micro-ecological regulation strategy towards the development of probiotics to ameliorate aquatic animal diseases.

Video Abstract

**Supplementary Information:**

The online version contains supplementary material available at 10.1186/s40168-023-01663-2.

## Introduction

The Pacific white shrimp (*Penaeus vannamei*) is the most widely cultured shrimp species, accounting for 70% of the total global shrimp production [1]. However, in the past decade, bacterial pathogens have caused considerable production losses and posed one of the biggest threats to the sustainability of the shrimp aquaculture industry [2]. At present, an emerging disease in shrimp-producing countries is acute hepatopancreatic necrosis disease (AHPND), which is mainly caused by the virulent *Vibrio parahaemolyticus* [3]. AHPND often occurs suddenly with high mortality at any point during the entire culture process and has seriously affected the yield and quality of shrimp culture, especially in Asia [4]. Various chemical bactericides and high-water exchange have been widely used for controlling AHPND. However, these measures have been criticized because they are a potential threat to human health and local ecosystems. Sustainable intensification in aquaculture depends on the development of practices that improve shrimp health without causing environmental disruption. Therefore, the development of an eco-friendly and economic alternative culture strategy is desperately needed to control diseases and improve shrimp survival.

Host-associated microbes play significant roles in host health, and a number of studies have revealed that the host microbiome could inhibit pathogen invasion and reduce disease occurrence [5]. Recently, microbiome interventions have been proposed as an alternative strategy for the prevention and control of disease outbreaks in shrimp aquaculture [6]. A successful practice for this involves the construction of a biofloc system (BFS), which works via supplementing carbon sources to stimulate the growth of heterotrophic bacteria [7]. Previous studies have indicated that a BFS can induce a more stable bacterial community that is well characterized by low within-group variation, a highly complex network and high robustness [8, 9]. The disruption of the gut bacterial community is related to infection by a number of shrimp pathogens, including *V. parahaemolyticus* [10, 11]. However, whether the stability of the bacterial community induced by a BFS is responsible for preventing the colonization and overgrowth of pathogens is less clear. Moreover, a BFS also induced the enrichment of core taxa belonging to the phylum Actinobacteria and the families Rhodobacteraceae and Flavobacteriaceae in shrimp guts [8]. These core taxa are closely associated with reduction in shrimp disease occurrence [8]. However, the causal relationship between these taxa enriched in a BFS and the shrimp resistance against pathogens remains to be fully clarified. This is essential information for constructing synthetic microbial communities (SynComs) for the maintenance of the healthy shrimp growth.

Fecal microbiota transplantation (FMT) has demonstrated potential in elucidating the physiological significance of the gut microbiome, especially in humans and wild mammals [12, 13]. Gut microbiota dysbiosis has been confirmed to contribute to shrimp white feces syndrome via the transplantation of the gut microbiota of the syndrome to healthy shrimp [14]. However, the complexity and variability of undefined-fecal microbiota also present great challenges for determining exactly which species are involved in a phenotype of interest [15]. For example, FMT only provides information on the final function of complete community, not the contributions of each taxa, such as the capacity to suppress pathogen colonization [16, 17]. The construction of SynComs provides a simplified approach to connect the composition of resident microbial communities with their potential function [16, 18]. However, two key limitations of SynComs construction are microbe isolation and optimization of the combinations of different strains [19]. These limitations emphasize the need for developing an efficient methodology for constructing a functional SynCom. Moreover, many studies have revealed that a community is much more likely to survive and function than a single strain in a non-sterile environment [20]. However, to what extent a SynCom stemmed from a local microbiome is effective at improving host health in a non-sterile environment remains unclear.

In this study, we hypothesized that the gut bacterial consortium induced by a BFS can improve shrimp health and disease resistance. The survival rates after *V. parahaemolyticus* infection, and the gut bacterial communities of shrimp cultured in a water exchange system (WES) and BFS were compared. The causal relationship between pathogen infection resistance and gut bacterial community was confirmed via the cross-transplantation of bacterial consortia enriched from WES and BFS shrimp. The underlying suppressive mechanisms of the gut bacterial consortium induced by the BFS were investigated from the aspects of bacterial community stability and immune activation. Finally, a disease-resistant SynCom was constructed to test its roles in specifically promoting shrimp growth and controlling disease occurrence in a non-sterile environment. The schematic diagram of the experimental design is shown in Figure S1.

## Materials and methods

### Shrimp source and culture

Two batches of juvenile Pacific white shrimp (*Penaeus vannamei*) at an initial average body weight of 3.0 g were obtained from local shrimp farms (Zhejiang, China), and cultured in two different culture systems: WES and BFS. Four batches of specific pathogen-free shrimp postlarvae (PL10 or PL26) were purchased from Hainan Haiyi Aquatic Seedlings Co. Ltd. (Wenchang, Hainan) or Hainan CPM Aquatic Puqian Co. Ltd. (Wenchang, Hainan), and used for the bacterial consortium addition experiments (Table S1). For the culture experiment, shrimp juveniles were farmed in 750-L canvas tanks containing 500 L of sanitized seawater with a density of 120 shrimp per tank for 3 weeks. For the bacterial consortia addition experiments, the shrimp postlarvae were cultured in 5-L plastic bottles containing 3.5 L of sanitized seawater with a density of 240 shrimp for 6 days. The shrimp were fed three times per day (at 08:00, 12:00, and 20:00), and the daily feeding was set as 4.5% of body weight. Constant aeration was provided in all experimental tanks or bottles using air stones during the entire experiment. All experiments were performed in a controlled growth chamber at 26 ± 2℃ in the light for 14 h and in the dark for 10 h at the pilot base of Ningbo University (Meishan Campus, Zhejiang, China).

### Experimental design and sampling

The first batch of juvenile shrimp was acquired from Jiaojiang Farm (Taizhou, Zhejiang) on November 8, 2021, and was acclimatized to the experimental condition for 3 days, and randomly distributed in 10 canvas tanks with a density of 120 shrimp per tank. Ten canvas tanks were randomly divided into two groups (five replicates per group): WES, fed on a basal diet (Zhejiang Qiangpu Biotechnology Co., Ltd., Zhejiang, China), and BFS, fed on a mixture of a basal diet and brown sugar (89.0%, w/w, Carbohydrate content) with a ratio of 3:4 to obtain a C/N ratio of ~ 10:1. A ~ 50% water exchange was performed in the WES to maintain normal shrimp growth, and no water exchange was carried out in the BFS. Bioflocs began to form after 1 week of cultivation, and biofloc volumes were maintained in the range of 20 − 30 mL L^−1^ after 2 weeks of cultivation. After 3 weeks of cultivation, the rearing water was first filtered through a 100-μm pore-sized filter to remove large-sized organic matter, and then through a 0.22-μm polycarbonate membrane to collect the water samples. Shrimp in each tank were harvested to calculate the growth parameters, and 10 shrimp from each tank were randomly chosen for sampling. Their guts and hepatopancreas were separated using sterile instruments to measure the gut bacterial communities and enzyme activities, respectively. The gut samples were uniformly mixed and divided into two parts. One part was homogenized in 1 mL of phosphate buffer solution (PBS) and stored with 25% glycerol (v/v) at − 80℃ for bacterial isolation and bacterial consortia enrichment. The other part was directly stored at − 80℃ for bacterial community analysis. Sampling was executed after a 24-h fasting period.

### Measurements of growth parameters and enzyme activities

Growth parameters such as survival rate, body weight, and body length were measured at the end of the experiment. Twenty shrimp were randomly harvested from each tank to determine their body weight (g) and body length (cm), respectively. Survival rates of shrimp in each tank were acquired by calculating the survival numbers at the end of the experiment. The activities of amylase (AMS), CMCase (carboxymethyl cellulase), lipase, acid phosphatase (ACP), alkaline phosphatase (AKP), catalase (CAT), superoxide dismutase (SOD), glutathione peroxidase, and total nitric oxide synthase, and the total anti-oxidative capacity were measured using their corresponding kits (Jiancheng Bioengineering Institute, Nanjing, China) according to the manufacturer’s instructions.

### Shrimp resistance against pathogenic Vibrio parahaemolyticus infection

The pathogenic *V. parahaemolyticus* FX1 strain was previously isolated from diseased shrimp with AHPND, and its pathogenicity was re-confirmed by an immersion challenge test using healthy shrimp according to the method of Restrepo et al. [21] with minor modifications. Briefly, the FX1 strain was cultured overnight in marine broth 2216E (MB; Becton–Dickinson) at 30℃. The fermentation broth was then pelleted (12,000 × *g* for 10 min) and washed twice in sterile seawater. Fifteen shrimp from each tank were distributed in a plastic box (67 × 45 × 35 cm) containing the FX1 resuspension solution at a concentration of 1.0 × 10^8^ colony-forming units (CFU) mL^−1^ with clean seawater for 15 min, and the concentration of pathogen was then diluted to 1.0 × 10^7^ CFU mL^−1^ through the addition of clean seawater. The shrimp were fed twice per day (at 08:00, and 17:00) and water exchange was not performed during the infection process. Mortality was monitored every 2 h until 40 h after infection. Another 15 shrimp from each tank that were not exposed to the pathogen resuspension solution were used as the negative control. At the end of experiment, the guts of shrimp that survived were collected and stored at − 80℃ for bacterial community analysis. The copy numbers of AHPND-related gene (*PirA*) were detected by quantitative reverse transcription polymerase chain reaction according to a previous study [11].

### Preparation of shrimp gut bacterial consortia and cross-transplantation

To enrich the shrimp gut bacterial community, the gut samples stored in the PBS were inoculated to the marine R2A liquid medium prepared with 75% seawater (salinity 30‰) and 25% distilled water according to the description by Li et al. [22]. An initial inoculum concentration of 0.1% was added into the R2A liquid medium and the mixture was incubated at 30℃, with agitation at 200 rpm for 36 h. The marine R2A medium is an oligotrophic medium that has been widely used to obtain more diverse heterotrophic bacteria from the marine environment [23] and mariculture animals [24, 25]. To reduce the heterogeneity, 10 replicates were performed to culture the bacterial consortia, which were combined together before the next step. The combined cultures were divided into two parts. One part was stored with 25% glycerol (v/v) at − 80℃ for isolation of bacteria and subculture. The other part was centrifuged at 12,000 × *g* for 10 min at 4℃ to collect the cell pellets, which were washed twice by sterilized PBS, and stored either at − 80℃ for bacterial community sequencing or 4℃ for cross-transplantation.

The cross-transplantation of bacterial consortium was carried out by the reverse gavage method described by Aranguren et al. [26]. Briefly, bacterial consortium suspensions from WES (Enrich^WES^) and BFS (Enrich^BFS^) shrimp were respectively adjusted to a concentration of optical density at 600 nm (OD_600_) = 2.0, and mixed with a commercial red food dye (Dongguan Jinjiahe Food Co., Ltd. Guangdong, China) at a ratio of 5000:1 (v/v). Shrimp in each tank from the above different culture systems (WES and BFS) were fasted for 24 h prior to cross-transplantation. Forty-five shrimp were randomly selected from each tank to perform the transplantation. Fifteen of these were used to transplant the bacterial consortium of shrimp from the same culture system, and in the other 15 shrimp, the bacterial consortium was transplanted from the different culture system. The remaining 15 shrimp were inoculated by reverse gavage using a 10 μL PBS as control. A 10-μL sample of bacterial consortium was slowly introduced to avoid any traumatic effects by using a sterile microsyringe. This resulted in six treatments: WES + PBS, WES + Enrich^WES^, WES + Enrich^BFS^, BFS + PBS, BFS + Enrich^WES^, and BFS + Enrich^BFS^, and each treatment included 75 shrimp. The shrimp were reared in three 100-L plastic boxes (67 × 45 × 35 cm) including 50 L of clean sanitized seawater in each box at the density of 25 shrimp. Twelve hours after the cross-transplantation, the shrimp were fed twice a day. After 36 h of the inoculation process, five shrimp from each tank were randomly harvested to collect their guts for bacterial community analysis. The remaining 20 shrimp were equally divided into two groups, the control group and pathogen infection group, according to the above description.

### Gut bacterial consortium addition in shrimp postlarvae

To perform the gut bacterial consortium addition experiment, the first batch of specific pathogen-free shrimp postlarvae (PL10) was purchased from Hainan Haiyi Aquatic Seedlings Co. Ltd. on May 13, 2022. The postlarvae were acclimatized to the experimental condition for 3 days prior to the experiment, and were then randomly stocked in each of the 18 5-L bottles containing 3.5-L clean sanitized seawater with a density of 240 shrimp. These tanks were randomly divided into three groups: control (eight replicates); Enrich^WES^ (five replicates), addition of the bacterial consortium enriched from WES shrimp; Enrich^BFS^ (five replicates), addition of the bacterial consortium enriched from BFS shrimp. The gut bacterial consortia were obtained by subculture in marine R2A medium for 36 h, then the cell pellets were collected by centrifuging at 12,000 × *g* for 10 min at 4℃, and washed twice in sterile seawater. The prepared bacterial consortia were poured into the bottles to reach a bacterial concentration of 1.0 × 10^7^ CFU mL^−1^ of water. A ~ 50% water exchange was performed every 2 days, and bacterial consortia were added again to reach a bacterial concentration of 1.0 × 10^7^ CFU mL^−1^ of water. The bacterial consortia were also added by mixing them with commercial shrimp flake daily for 10 min at the concentration of 1.0 × 10^7^ CFU g^−1^ of feed, and immediately administrated at shrimp feeding. After 6 days of culture, the numbers of shrimp that survived in each bottle were recorded to calculate the survival rate, and the values of gut fullness were obtained by calculating the ratio of gut length to body length. Ten shrimp were randomly harvested from each bottle and were rinsed twice by 75% ethanol, followed by sterile seawater three times, and stored at − 80℃ for bacterial community analysis. Pathogen infection experiment was carried out in a 650-mL glass bottle containing 500 mL of clean seawater with a density of 25 shrimp, and 15 replicates were included in each treatment. The survival rates of shrimp were recorded after 48 h of infection.

### Histopathology analysis

Gut tissues of adult shrimp or whole postlarvae were fixed in 4% paraformaldehyde solution at 4℃ for 24 h, and then transferred to 70% ethanol. The fixed samples were dehydrated in a graded series of ethanol and xylene, and embedded in paraffin [27]. The paraffin blocks were sectioned at a thickness of 5 µm using a Leica RM2135 rotary microtome, stained with hematoxylin/eosin, and observed using a Zeiss LSM780 Laser Scanning Confocal Microscope (Carl Zeiss SAS, Jena, Germany).

### Reproducibility of gut bacterial consortium-induced Vibrio infection resistance in the BFS

To evaluate the reproducibility of the gut bacterial consortium of shrimp cultured in the BFS against pathogen infection, the shrimp were cultured again in different culture systems and their gut bacterial consortia were enriched to measure the resistance against *Vibrio* infection. The second batch of juvenile shrimp at an initial weight of ~ 3 g each were acquired from Maoyang Farm (Ningbo, Zhejiang) on May 13, 2022, and were cultured in the WES and BFS with six replicates each. After 3 weeks of culture, direct *Vibrio* infection or infection after the cross-transplantation of bacterial consortia was performed according to the above description. The second batch of specific pathogen-free postlarvae (PL10) was bought from Hainan Haiyi Aquatic Seedlings Co. Ltd. on June 21, 2022, to carry out the bacterial consortia addition experiment.

### DNA extraction and 16S rRNA gene sequencing

Bacterial DNA was extracted from water sample and enrichment culture using a Power Soil® DNA isolation kit (MOBIO, USA), and from gut and whole postlarvae sample using a QIAamp® DNA Stool Mini Kit (Qiagen, Germany) following the manufacturer’s instructions, respectively. The concentration and quality of DNA were measured using a Nanodrop 2000 spectrophotometer (NanoDrop Technologies, Wilmington, DE, USA), and DNA was stored at − 20 °C for further analysis. The V4 region of the 16S rRNA gene was amplified using primers 515F-Y (5′-GTGYCAGCMGCCGCGGTAA-3′) and 806R (5′-GGACTCANVGGGTWTCTAAT-3′). The sequencing was performed on the Illumina MiSeq platform (Illumina, USA).

### Sequencing data analysis

Raw data were processed and analyzed using Quantitative Insights into Microbial Ecology (QIIME) 1.9.1 (http://qiime.org/) [28]. Quality control and chimera removal of sequences were performed using USEARCH [29]. The remaining sequences were clustered into operational taxonomic units (OTUs) at 99% sequence similarity cutoff using the *pick_open_reference_otus.py* script. A representative sequence for each OTU was annotated taxonomically based on the SILVA 128 database, and all singletons and OTUs associated with chloroplasts or mitochondria, and all unassigned and unclassified sequences were removed from the data. Alpha-diversity indexes were computed using QIIME 1.9.1, and the variable coefficient was obtained by dividing the standard deviation by the mean value within groups. The differences in bacterial community among groups were shown by principal coordinate analysis (PCoA), and analysis of similarity based on the Bray − Curtis (BC) dissimilarity. SourceTracker analysis was used to evaluate the migration of gut microbiota [30]. Differentially abundant OTUs were identified at false discovery rate corrected *p* < *0.05* between treatments using the “DESeq2” package in R 4.0.3 [31]. Microbes with average reads ≥ 5 were selected to construct the interaction network using SparCC, and a median of 20 and 100 bootstraps were used to calculate robust correlations and pseudo-*p*-values using the “SpiecEasi” package in R 4.0.3 [32]. Networks were produced by retaining the robust correlations (|ρ|≥ 0.6, *p* < *0.05*), and visualized with Gephi 0.9.2 [33]. The network stability parameters including cohesion value and robustness were estimated according to a previous study [8].

### Bacterial isolation from shrimp guts and gut bacterial consortia

To isolate bacterial strains, serial dilutions of the glycerol stocks obtained from shrimp guts and enrichment culture were plated on R2A, 1/2 marine 2216E, and 1/10 marine 2216E agar media. Plates were incubated at 28℃ for 2 weeks, and colonies of bacteria showing different morphological features, such as size and color, were re-streaked on marine 2216E plate to ensure purity. The genome of the purified strain was extracted by 10% Chelex solution, and the 16S rRNA full-length sequence was amplified using the universal primers F27 (5′-AGAGTTTGATCMTGGCTCAG-3′) and R1492 (5′-TACGGYTACCTTGTTACGACTT-3′), and purified for sequencing. These sequences were then submitted to EZ_BioCloud_ database for taxonomic identification (https://www.ezbiocloud.net/identify). Based on the gut bacterial community compositions of BFS shrimp and Enrich^BFS^, a total of 250 strains belonging to five candidate genera—*Paracoccus*, *Ruegeria*, *Microbacterium*, *Tenacibaculum*, and *Demequina*—were isolated.

To find the corresponding strains of enriched OTUs in the BFS and Enrich^BFS^, the V4 region of the 16S rRNA gene sequences from these isolated strains were aligned with the consensus sequences of the enriched OTUs. Finally, five representative strains assigned to the above genera, which showed more than 99% similarity with the V4 region of the enriched OTUs, were selected for potential function analyses and synthetic bacterial community construction. Whole-genome sequencing of these five strains was performed on the Illumina HiSeq PE150 platform. The production of protease, CMCase, and AMS were assayed by observing whether a halo was formed in the agar plate using 0.5% casein, CMC, and soluble starch, respectively, as the sole nitrogen source or carbon source. Biofilm and siderophore production were measured by the crystal violet assay [34] and chrome azurol S agar plate assay [35], respectively. The bacteriostatic activity of these isolates was investigated against *V. parahaemolyticus* FX1 by a dual culture method.

### Construction of SynCom against Vibrio infection

A synthetic community that included all five strains was constructed as SynCom^BFS^. Each strain was cultured separately in marine 2216E medium for 24 h, and the cell pellet was collected by centrifuging at 12,000 × *g* for 10 min at 4℃, and washed twice by sterile seawater. The resuspension solution of each strain was then adjusted to a final OD_600_ value of 1.0, and the SynCom^BFS^ was constructed by equal volume mixing of the suspension of each strain. The single strain or SynCom^BFS^ was added into the rearing water at a final concentration of 1.0 × 10^7^ CFU mL^−1^ of water, and simultaneously mixing with the feed at the concentration of 1.0 × 10^7^ CFU g^−1^ of feed. The shrimp that did not add any strain and the shrimp that added the Enrich^BFS^ were used as an inoculum-free control and positive control, respectively. The postlarvae (PL10, the third batch) were acquired from Hainan CPM Aquatic Puqian Co. Ltd. on September 10, 2022. After 6 days of addition, the resistance of shrimp against *Vibrio* infection was evaluated.

### Effect of SynCom^BFS^ on shrimp health during the entire culture process

The fourth batch of postlarvae (PL26, ~ 0.25 g each) was acquired from Hainan CPM Aquatic Puqian Co. Ltd. on October 11, 2022. The postlarvae were firstly acclimatized to the experimental condition for 3 days and were then randomly stocked in six 750-L canvas tanks containing 400 L of sanitized seawater with a density of 400 shrimp. These tanks were randomly divided into two groups (three replicates per group): CK, no SynCom addition; and SynCom^BFS^, addition of SynCom^BFS^. The strains from the SynCom^BFS^ were separately cultured in the marine 2216E for 24 h, and the OD_600_ values of each culture were adjusted to 1.0 before mixing them together in equal volume. The mixture were then added into the rearing water at a final concentration of 1.0 × 10^5^ CFU mL^−1^ of water, and re-supplied at the same concentration after water exchange. The SynCom^BFS^ was also added by feeding at the concentration of 1.0 × 10^7^ CFU g^−1^ of feed. The water was not exchanged for the first week in all tanks, and exchanged once every 3 days after 1 week. A ~ 15% water exchange was performed each time in the second week, 25% in the third week, and 50% from the fourth week until the end of the experiment. After 55 days of culture, shrimp in the CK group showed the usual clinical and pathological symptoms of *Vibrio* disease, such as lethargy, empty gut, and pale hepatopancreas, and started to die, while the shrimp in the SynCom^BFS^ group were always healthy. At 56 days of culture, the survival rates and growth parameters including body weight and length were recorded in all tanks. The shrimp in the SynCom^BFS^ group were continually cultured, and no disease occurred during the culturing period, which ended with the harvesting of shrimp at the average yield of 3.4 kg each tank at 80 days of culture.

To test whether the SynCom^BFS^ addition could rescue diseased adult shrimp in the control group, the shrimp that survived in each tank of the control group were randomly divided into three groups: CK; SynCom^BFS^, addition of SynCom^BFS^; Enrich^BFS^, addition of the Enrich^BFS^. In each group, 10 healthy shrimp and 10 diseased shrimp were included. The survival rates and the ratios of diseased shrimp were record at 3, 6, and 8 days after treatment. In addition, the postlarvae from the third batch in one tank exhibited serious illness after 33 days of culture, which caused approximately 90% shrimp mortality. The shrimp (~ 0.45 g each) were also randomly divided into three groups to test the recovery effect of SynCom^BFS^ addition on diseased shrimp. The survival rates and the ratios of diseased postlarvae were record at 1, 2, 3, and 4 days after treatment.

### Statistical analyses

Statistical analyses were performed to assess the significant differences between groups based on Student’s *t* test using the “VEGAN” package in R 4.0.3. The linear relationship and regression model between bacterial community stability parameters and *Vibrio* infection resistance were evaluated using the “lm()” R function.

## Results

### Gut bacterial consortium of BFS shrimp confers Vibrio infection resistance

To investigate the differences in the *Vibrio* infection resistance of shrimp in the various culture systems, shrimp were first cultured in the WES and BFS and were then infected by pathogenic *V. parahaemolyticus* (Figure S2a). The BFS shrimp showed better growth with higher survival rate, body weight, and body length compared with WES shrimp after 21 days of culture (Figure S2b). The BFS shrimp had a stronger resistance against *V. parahaemolyticus* infection than WES shrimp, with a higher survival rate and a longer duration until mortality (Figure S2c). The WES shrimp also showed more serious symptoms, such as empty intestines, lethargy, and hepatopancreas atrophy after infection than BFS shrimp (Figure S2d). The copies of virulence gene *PirA* were significantly lower in BFS shrimp guts, compared with WES shrimp (Figure S2f). Regarding the histopathological examination, BFS shrimp had a higher mucosal fold height of the midgut than WES shrimp before infection, and retained these complete structures after infection, while the midgut of WES shrimp exhibited epithelial cell detachment, disappeared microvilli, and a thinner muscle layer (Figure S2e). In addition, the enzyme activities of AMS, CMCase, ACP, AKP, CAT, and SOD were significantly increased in BFS shrimp, compared with WES shrimp (Figure S3), indicating that the BFS might improve the activities of digestive enzymes and immunoenzymes to resist *V. parahaemolyticus* infection.

**Fig. 1:**
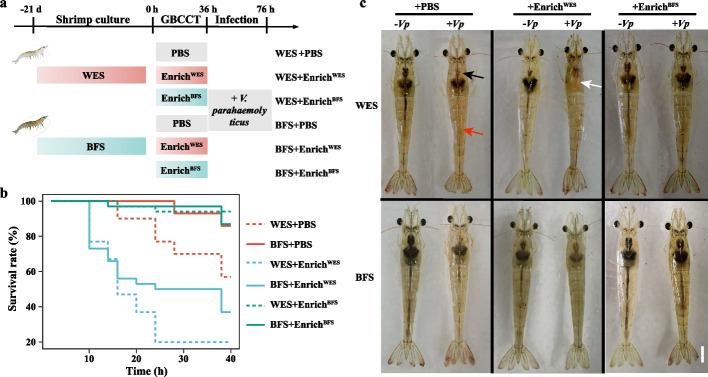
The *Vibrio* infection resistance, and phenotypic characteristics of shrimp after cross-transplantation of bacterial consortia enriched from WES and BFS shrimp. **a** Schematic diagram of the experimental procedure. The bacterial consortia were obtained from the shrimp cultured in the WES and BFS for 21 days, and the bacterial consortia were cross-transplanted to WES and BFS shrimp. After 36 h of cross-transplantation, the shrimp that received PBS and bacterial consortia were infected by the pathogenic *Vibrio parahaemolyticus*. **b** The effects of cross-transplantation on the shrimp survival curves after infection. **c** Phenotypic characteristics of shrimp after infection. Black, white, and red arrows indicate the stomach, hepatopancreas, and gut of shrimp in (**c**), respectively. Bar = 1 cm in (**c**). WES, Water exchange system; BFS, Biofloc system; PBS, Phosphatic buffer solution; Enrich^WES^, Bacterial consortium obtained from WES shrimp; Enrich^BFS^, Bacterial consortium obtained from BFS shrimp; − *Vp*, non-*Vibrio* infection; + *Vp*, *Vibrio* infection

To confirm whether the *V. parahaemolyticus* infection resistance of BFS shrimp was attributed to the gut bacterial community, a cross-transplantation of gut bacterial consortium was performed (Fig. 1a). The shrimp that received a transplantation of the gut bacterial consortium from WES shrimp (Enrich^WES^) were more susceptible to *V. parahaemolyticus* infection and stared to die at 10 h of infection, regardless of whether they were farmed in the WES or BFS. In contrast, the shrimp that received a transplantation of the gut bacterial consortium from BFS shrimp (Enrich^BFS^) showed greater resistance to *V. parahaemolyticus* infection, even if they were previously farmed in the WES, and still had a 90% survival rate after 40 h of infection (Fig. 1b). Shrimp phenotypes also indicated that WES shrimp in the Enrich^WES^ transplantation group had the most serious symptoms after 40 h of infection, with empty intestines, and hepatopancreas erosion, while the shrimp in the Enrich^BFS^ transplantation group maintained better intestines and hepatopancreas after *V. parahaemolyticus* infection, especially BFS shrimp that received Enrich^BFS^ (Fig. 1c). This suggests that the gut bacterial consortium of BFS shrimp can resist *V. parahaemolyticus* infection.

### BFS shrimp possess a more stable and resistant gut bacterial community

**Fig. 2:**
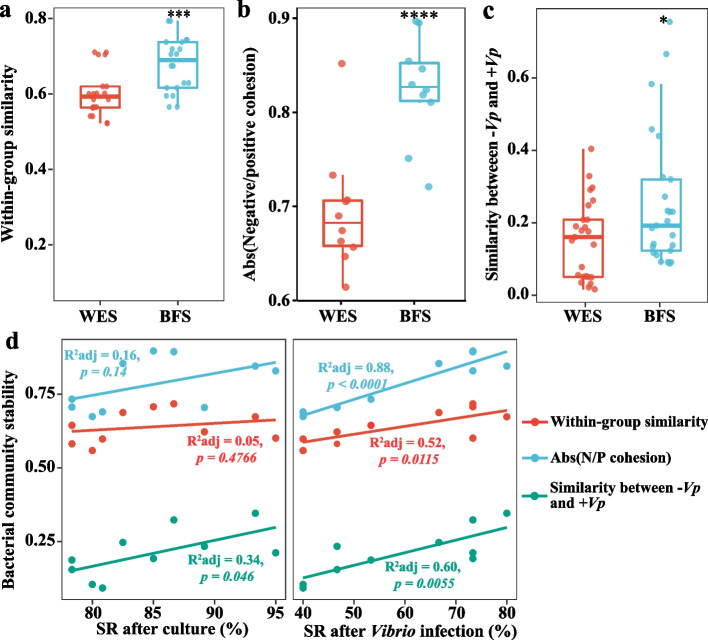
The gut bacterial community stability parameters of WES and BFS shrimp and its relationships with survival rate. **a** The within-group similarities of gut bacterial community in WES and BFS shrimp. **b** The absolute value of negative:positive cohesion ratio (N/P cohesion) of bacterial networks. **c** Bacterial community similarity between non-*Vibrio* infection and *Vibrio* infection groups in WES and BFS shrimp. The boxes represent the median and interquartile range, whiskers range from minimum to maximum values, and error bars show standard deviation. Each sample had five replicates (*n* = 5). The asterisk indicates a significant difference at *p < 0.05, ***p < 0.001, and ****p < 0.0001 based on Student’s *t* test. **d** The linear relationships of bacterial community stability parameters and survival rates after culture experiment and *Vibrio* infection. Adjusted R^2^ values and the significance levels are displayed for each of the linear regressions. WES, Water exchange system; BFS, Biofloc system; − *Vp*, non-*Vibrio* infection; + *Vp*, *Vibrio* infection; SR, Survival rate

To characterize the *V. parahaemolyticus* infection-resistant bacterial community, the gut bacterial community was measured by 16S rRNA amplicon sequencing. Although α-diversity indexes showed no significant differences (Student’s *t* test *p* > 0.05) between the WES and BFS in both water and shrimp gut samples, the variations in these indexes within the BFS group were remarkably reduced, compared with the WES group (Figure S4a and b), suggesting that the BFS might enhance the bacterial community stability in the water and gut. The bacterial community structures of the WES and BFS in both the water and shrimp gut samples were significantly different (*p* < 0.001) based on the PCoA analysis (Figure S4c), and the gut bacterial community showed a greater similarity with the water bacterial community in the BFS than in the WES (Figure S4d). Meanwhile, a larger gut bacterial community was sourced from the water in the BFS than in the WES, indicating that the BFS reshaped the gut bacterial community via the water bacterial community (Figure S4e). The BFS significantly enhanced the within-group BC-similarity of the bacterial community in both the water and gut samples (Fig. 2a; Student’s *t* test *p* < *0.05*), demonstrating that the BFS improved the bacterial community stability.

The bacterial community compositions differed in the various culture systems (Figure S4f). The BFS increased the relative abundances of taxa belonging to Rhodobacteraceae, Flavobacteriaceae, and Microbacteriaceae in both water and gut samples, and decreased the relative abundances of bacteria assigned to Vibrionaceae, Pseudoalteromonadaceae, and Pseudomonadaceae (Figure S4f). To further identify the microbe-microbe interactions in the WES and BFS, bacterial co-occurrence networks were constructed using SparCC (Figure S5a). The BFS greatly increased the complexity of co-occurrence networks with more nodes and edges, compared with the WES (Figure S5a). The modularity of the bacterial community network was enhanced in the BFS with a value of 33.88, compared with a value of 1.809 in the WES (Figure S5a), indicating that the bacterial community in the BFS was more compartmentalized than that in the WES. The bacterial networks of the BFS had more nodes belonging to gram-positive bacteria such as Actinobacteria and Firmicutes and possessed fewer nodes assigned as Proteobacteria and Bacteroidetes (Figure S5b). The average degree, closeness centrality, and betweenness centrality were increased in the BFS, compared with the WES, especially the betweenness centrality, which was significantly enhanced in the BFS (Figure S5c; Student’s *t* test *p* < 0.001). The ratio of negative:positive cohesion in the BFS was markedly increased due to the significant decrease in positive cohesion (Figs. 2b and S5d; Student’s *t* test *p* < 0.05). The robustness of the bacterial networks was always high when the same number of nodes was randomly removed in the BFS, compared with the WES (Figure S5e), indicating that the roles of the nodes in maintaining the stability of bacterial networks were enhanced in the BFS.

To further investigate the bacterial community resistance against pathogen infection, the BC-similarity of the gut bacterial community was compared between the non-*Vibrio* infection group and *Vibrio* infection group. Although the gut bacterial community structures in both WES and BFS shrimp were significantly altered after *V. parahaemolyticus* infection, the variability in BFS shrimp was remarkably smaller than that in WES shrimp (Fig. 2c; Student’s *t* test *p* < *0.05*). This indicates that the gut bacterial community was more resistant in the BFS than that in the WES when exposed to *V. parahaemolyticus* infection. Furthermore, the stability parameters, such as bacterial community within-group similarity, ratio of negative:positive cohesion, and bacterial community similarity between non-*Vibrio* and *Vibrio* infection, were strongly and positively correlated with the survival rate of shrimp, especially the survival rate after *V. parahaemolyticus* infection (Fig. 2d), implying that the gut bacterial community stability induced by the BFS might increase the resistance of shrimp against pathogen infection.

### Transplantation of a gut bacterial consortium from BFS shrimp promotes the stability and resistance of gut bacterial community

**Fig. 3:**
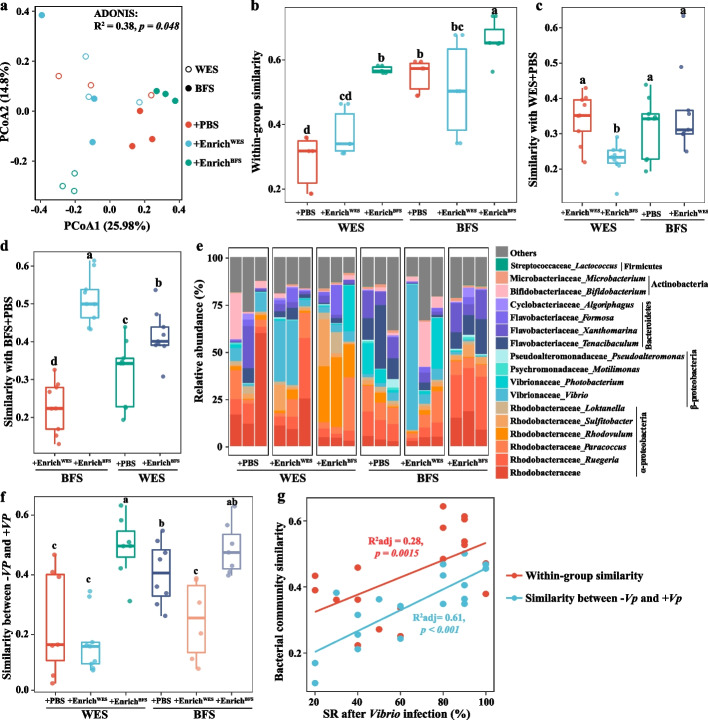
The gut bacterial community characteristics of shrimp after cross-transplantation of gut bacterial consortia. **a** Principal coordinate analysis (PCoA) plots based on the Bray–Curtis dissimilarity. **b** The within-group similarities of bacterial community of shrimp gut samples after cross-transplantation. **c** The bacterial community similarities of WES + PBS and other groups. **d** The bacterial community similarities of BFS + PBS and other groups. **e** The bacterial community compositions after cross-transplantation. **f** The gut bacterial community similarities of shrimp between non-*Vibrio* infection and *Vibrio* infection. The boxes represent the median and interquartile range, and whiskers range from minimum to maximum values. Each sample had three replicates (*n* = 3). Different letters indicate a significant difference at* p* < 0.05 based on Student’s *t* test. **g** The linear relationships of bacterial community similarity and survival rates after *Vibrio* infection. Adjusted R^2^ values and the significance levels are displayed for each of the linear regressions. WES, Water exchange system; BFS, Biofloc system; PBS, Phosphatic buffer solution; Enrich^WES^, Bacterial consortium obtained from WES shrimp; Enrich^BFS^, Bacterial consortium obtained from BFS shrimp; − *Vp*, non-*Vibrio* infection; + *Vp*, *Vibrio* infection; SR, Survival rate

Given the different roles of Enrich^WES^ and Enrich^BFS^ after transplantation against *V. parahaemolyticus* infection, their bacterial community compositions were characterized (Figure S6). Although the bacterial compositions of Enrich^WES^ and Enrich^BFS^ mainly comprised *Vibrio*, with relative abundances of 89.5% and 84.4% in Enrich^WES^ and Enrich^BFS^, respectively (Figure S6a), the relative abundances of six OTUs from the following genera—*Tenacibaculum*, *Lactococcus*, *Paracoccus*, *Microbacterium*, *Ruegeria*, and *Demequina*—were significantly higher in Enrich^BFS^ than in Enrich^WES^ (Figure S6b; Student’s *t* test* p* < 0.05). To assess whether the *V. parahaemolyticus* infection resistance was associated with the gut bacterial community stability after cross-transplantation, gut bacterial communities of shrimp that received transplantation were measured (Fig. 3). The bacterial community structures were significantly altered after the transplantation of the gut bacterial consortia (Fig. 3a; ADONIS: *p* = 0.048). Although shrimp that received an Enrich^WES^ transplantation did not significantly change the within-group BC-similarity, it increased the within-group bacterial community variation, compared with shrimp that received PBS, especially when Enrich^WES^ was transplanted into BFS shrimp (Fig. 3b). In contrast, shrimp that received an Enrich^BFS^ transplantation not only significantly enhanced the within-group BC-similarity, but also showed a larger reduction in the within-group bacterial community variation, regardless of whether the shrimp were previously farmed in the WES or BFS (Fig. 3d; Student’s *t* test *p* < 0.05). This indicated that transplantation of Enrich^BFS^ improved the stability of gut bacterial community. Moreover, the BC-similarity of the bacterial community between WES + PBS and WES + Enrich^BFS^ was significantly lower (Student’s *t* test *p* < *0.05*) than that between WES + PBS and WES + Enrich^WES^, while BFS + Enrich^WES^ did not significantly change the BC-similarity of the bacterial community with WES + PBS, compared with WES + PBS and BFS + PBS (Fig. 3c, d). Of note, shrimp that received an Enrich^BFS^ transplantation remarkably elevated the BC-similarity of bacterial community with BFS + PBS, regardless of whether the shrimp were previously farmed in the WES or BFS (Fig. 3d; Student’s *t* test *p* < *0.05*).

Regarding the gut bacterial community compositions, the transplantation of Enrich^WES^ mainly increased the relative abundance of *Vibrio*, and reduced the relative abundances of taxa from Rhodobacteraceae in both WES and BFS shrimp, compared to their respective controls (Fig. 3e). The transplantation of Enrich^BFS^ into WES and BFS shrimp greatly enhanced the relative abundances of taxa from Rhodobacteraceae (mainly *Rhodovulum* in the WES, and *Ruegeria* in the BFS), Flavobacteriaceae and *Microbacterium*, and decreased the relative abundance of *Vibrio* (Fig. 3e). Moreover, the bacterial compositions were more stable when the shrimp received a transplantation of Enrich^BFS^, compared with PBS or Enrich^WES^, regardless of whether the shrimp were previously farmed in the WES or BFS (Fig. 3e). The BC-similarities of the bacterial community between the non-*Vibrio* infection group and *Vibrio* infection group in WES + Enrich^BFS^ and BFS + Enrich^BFS^ were significantly higher (Student’s *t* test *p* < *0.05*) than that in Enrich^WES^-transplanted shrimp (Fig. 3f). This further confirms that the bacterial community in the BFS had a stronger resistance against *V. parahaemolyticus* infection than in the WES. Furthermore, the within-group similarity, and similarity between non-*Vibrio* and *Vibrio* infection of bacterial community were strongly and positively correlated with the survival rate after *V. parahaemolyticus* infection (Fig. 3g), further indicating that the gut bacterial community stability was responsible for the pathogen resistance of shrimp.

### Gut bacterial consortia addition enhances Vibrio infection resistance in shrimp postlarvae

**Fig. 4:**
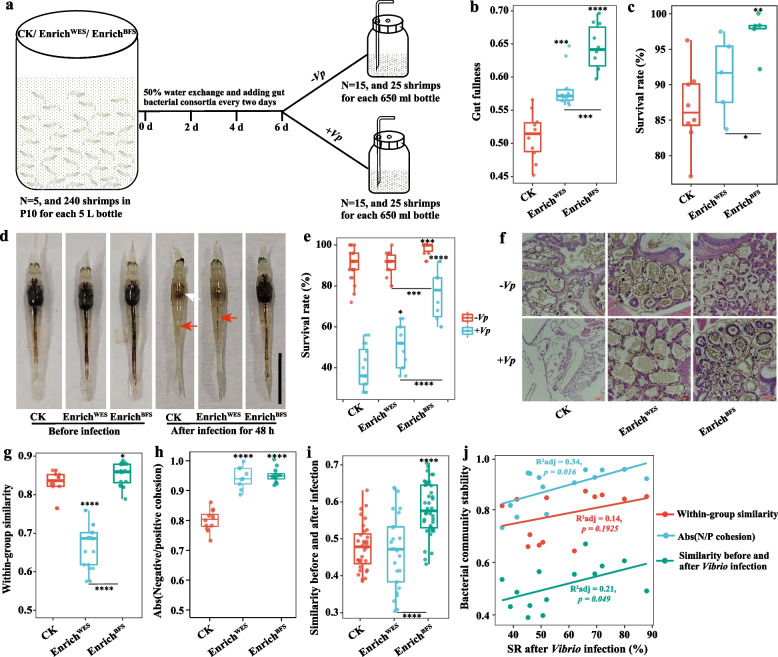
Effects of bacterial consortia addition on the phenotypic characteristic, *Vibrio* infection resistance, and bacterial community of shrimp postlarvae. **a** Schematic diagram of the experimental procedure. The bacterial consortia were added to the postlarvae system every 2 days, and the shrimp were infected by the pathogenic *Vibrio parahaemolyticus* after 6 days of culture. **b** Gut fullness. **c** Survival rate after bacterial consortia addition for 6 days. **d** Phenotypic characteristics of postlarvae after bacterial consortia addition and *Vibrio* infection. White and red arrows indicate the hepatopancreas and gut of shrimp, respectively. Bar = 1 cm. **e** Survival rate of shrimp after *Vibrio* infection. **f** Histological sections of hepatopancreas from postlarvae. **g** The within-group similarities of bacterial community. **h** The absolute value of negative:positive cohesion ratio (N/P cohesion) of bacterial networks. **i** The bacterial community similarities before and after *Vibrio* infection in the control and bacterial consortia addition groups. The boxes represent the median and interquartile range, and whiskers range from minimum to maximum values. Different asterisks indicate a significant difference at **p < 0.01, ***p < 0.001, and ****p < 0.0001 based on Student’s *t* test. **j** The linear relationships of bacterial community stability parameters and survival rates after *Vibrio* infection. Adjusted R^2^ values and the significance levels are displayed for each of the linear regressions. WES, Water exchange system; BFS, Biofloc system; PBS, Phosphatic buffer solution; Enrich^WES^, Bacterial consortium obtained from WES shrimp; Enrich^BFS^, Bacterial consortium obtained from BFS shrimp; − *Vp*, non-*Vibrio* infection; + *Vp*, *Vibrio* infection

To evaluate whether the gut bacterial consortia can be used as a probiotic to increase resistance against *V. parahaemolyticus* infection, gut bacterial consortia were added into the postlarvae culture system (Fig. 4a and S7). The gut fullness, defined as the ratio of gut to body length, was significantly enhanced by 14.2 and 26.4% with the addition of Enrich^WES^ and Enrich^BFS^, respectively, compared with the control group (Fig. 4b and S7c; Student’s *t* test *p* < 0.001). The 96.4% survival rate of the Enrich^BFS^ addition group was higher than that of the control and Enrich^WES^ addition groups after 6 days of culture (Fig. 4c). The addition of gut bacterial consortia improved the vitality and digestive system of shrimp, compared with the control group (Fig. 4d). The symptoms of shrimp in the Enrich^BFS^ addition group were obviously milder than those in the control and Enrich^WES^ addition groups after 48 h of infection (Fig. 4d). The shrimp in the Enrich^BFS^ addition group maintained an intact stomach and hepatopancreas and had better intestines, while the hepatopancreas and intestines of shrimp in the control and Enrich^WES^ addition groups were eroded or even disappeared, especially in control group (Fig. 4d). After *V. parahaemolyticus* infection, the survival rate of shrimp in Enrich^BFS^ addition group was remarkably increased by 90.0 and 51.5%, respectively, compared to that in the control and Enrich^WES^ addition groups (Fig. 4e; Student’s *t* test *p* < *0.001*). The hepatopancreas morphology results showed that the numbers of secretory cells (B-cells) were greatly increased, and the hepatopancreatic tubules were more tightly arranged in the Enrich^BFS^ addition group. After *V. parahaemolyticus* infection, the hepatopancreas morphology was completely destroyed in the control group, and the hepatopancreatic tubules and basal membrane were broken in the Enrich^WES^ addition group (Fig. 4f). However, the hepatopancreatic tubules were still closely arranged and the basal membrane was well retained in the Enrich^BFS^ addition group (Fig. 4f). Moreover, the addition of Enrich^BFS^ significantly increased the enzyme activities of CMCase, ACP, AKP, CAT, and SOD, compared with the control group (Figure S8; Student’s *t* test* p* < 0.05).

The addition of gut bacterial consortia significantly changed the bacterial community structure and composition of shrimp. The bacterial richness, evenness, and phylogenetic diversity were remarkably increased in both Enrich^WES^ and Enrich^BFS^ addition groups, compared with the control group (Figure S9a; Student’s *t* test *p* < 0.001). The within-group BC-similarity of the bacterial community was significantly increased (Student’s *t* test *p* < 0.05) in the Enrich^BFS^ addition group, while it was greatly reduced (Student’s *t* test* p* < 0.001) in the Enrich^WES^ addition group, compared with the control group (Fig. 4g). Meanwhile, the BC-similarity between the Enrich^BFS^ addition and control groups was significantly higher than that between the Enrich^WES^ addition and control groups before infection, while it was significantly decreased after *Vibrio* infection (Figure S9b and c; Student’s *t* test *p* < 0.01). These results indicate that the Enrich^BFS^ addition might increase the stability of the bacterial community through small changes in the bacterial community structure, while Enrich^WES^ addition might cause larger perturbations in the microbial community.

The addition of gut bacterial consortia mainly enhanced the relative abundances of Flavobacteriaceae such as *Tenacibaculum*, *Ruegeria*, and *Microbacterium* (Figure S9d). Furthermore, the addition of the gut bacterial consortia also increased the complexity of the bacterial community networks of shrimp postlarvae through enhancement of the nodes belonging to Bacteroidetes, Firmicutes, and Actinobacteria, node degree, closeness centrality, and betweenness centrality (Figure S10a-c). The stability parameters of the bacterial community networks, such as modularity, cohesion, and robustness were significantly increased in the Enrich^BFS^ addition group (Figs. 4h and S10a, d, e; Student’s *t* test *p* < *0.01*), further suggesting that Enrich^BFS^ addition might help to maintain the stability of bacterial networks. The BC-similarity of the bacterial community before and after infection was significantly higher in the Enrich^BFS^ addition group than that in the control and Enrich^WES^ addition groups, and showed a significant positive correlation with survival rate of shrimp after infection (Fig. 4i, j; Student’s *t* test *p* < *0.05*). This indicates that Enrich^BFS^ addition could also improve resistance against *Vibrio* infection through enhancing the gut bacterial community stability of shrimp.

### BFS-enriched bacterial consortium can stably promote the resistance of shrimp against *V. parahaemolyticus* infection

In order to confirm that *V. parahaemolyticus* infection resistance is promoted by the bacterial consortium enriched in the BFS, another batch of shrimp was cultured in the BFS (Figures S11 and S12). The survival rate of shrimp cultured in the BFS was significantly higher than that in the WES even though the batch of shrimp and culture season differed (Figure S11a; Student’s *t* test *p* < *0.05*). Although the gut bacterial community compositions differed between the first and second culture experiment, shrimp cultured in the BFS all significantly induced the enrichment of *Paracoccus*, *Ruegeria*, *Tenacibaculum*, *Microbacterium*, and *Demequina*, compared with the WES (Figure S11b). A cross-transplantation experiment was then performed using the gut bacterial consortia from the second passage in the first culture experiment (Enrich^WES_PN2^ and Enrich^BFS_PN2^) and the gut bacterial consortia from the first passage in the second culture experiment (Enrich^WES_PN1^ and Enrich^BFS_PN1^) (Figure S12a). The survival rates of Enrich^BFS_PN2^ and Enrich^BFS_PN1^-transplanted shrimp were significantly enhanced (Student’s *t* test, *p* < 0.05) after *V. parahaemolyticus* infection (Figure S12b). Furthermore, the addition of Enrich^BFS_PN2^ and Enrich^BFS_PN1^ into the postlarvae culture system also increased the resistance to *V. parahaemolyticus* infection (Figure S12c and d). These results indicate that the gut bacterial community of shrimp cultured in the BFS stably enhanced their resistance against *V. parahaemolyticus* infection.

### SynCom^BFS^ protects shrimp against *V. parahaemolyticus* infection under non-sterile conditions

Of the bacterial taxa significantly enriched in the BFS and gut bacterial consortium, five genera—*Paracoccus*, *Ruegeria*, *Microbacterium*, *Tenacibaculum*, and *Demequina*—were selected as the potential *Vibrio*-resistant taxa (Table S2). To further characterize these genera, the corresponding microbes were isolated from the shrimp gut and bacterial consortium. A total of 250 bacterial strains were isolated and identified as the above five genera based on 16S rRNA gene sequences (Table S3). The V4 region of the 16S rRNA gene sequence from each isolate was then mapped back to an OTU with the highest relative abundance in each of the above genera. Ultimately, five representative strains for the above genera could be screened with more than 99% similarity between them and were used in the further studies (Table S4). All five strains belong to undescribed species or species for which the type strain has not been completely sequenced based on the digital DNA-DNA hybridization and average nucleotide identity through estimating the genomic distances with the most similar type strains (Table S4). All strains showed excellent digestive enzyme activities with protease production in all strains or CMCase and AMS production in the partial strains. The five strains all revealed the ability of biofilm production, especially *Paracoccus* sp. RBB26, *Tenacibaculum* sp. HBG23, and *Demequina* sp. TCG4, of which the OD_590nm_ values all exceeded 4.5 (Table S5). Furthermore, the acid-producing, siderophore-producing and antagonistic pathogenic bacteria abilities of these strains were measured, but the results were negative (Table S5).

**Fig. 5:**
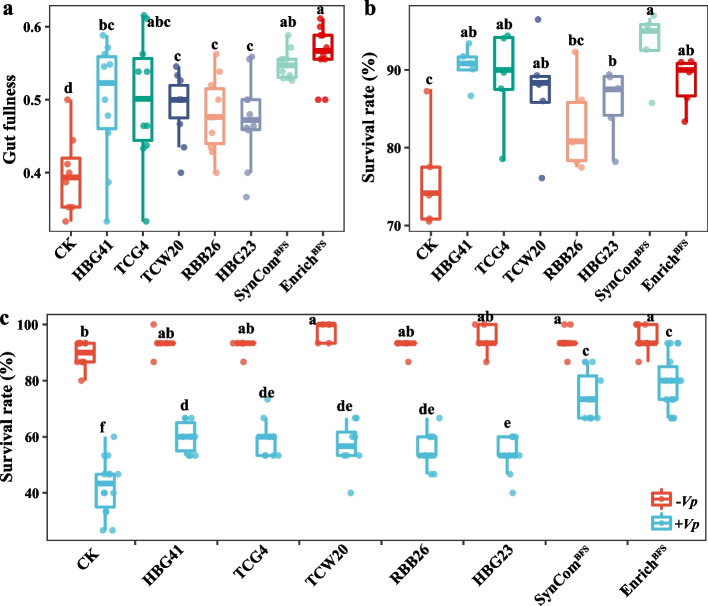
Effects of individual strains and SynCom^BFS^ addition on the growth characteristics, *Vibrio* infection resistance of shrimp postlarvae after 6 days of culture. **a** Gut fullness (*n* = 15). **b** Survival rate of postlarvae after 6 days of culture (*n* = 5). **c** Survival rates of postlarvae after *Vibrio* infection (*n* = 15). The boxes represent the median and interquartile range, and whiskers range from minimum to maximum values. Different letters indicate a significant difference at *p* < 0.05 based on Student’s *t* test. SynCom, Synthetic community; − *Vp*, non-*Vibrio* infection; + *Vp*, *Vibrio* infection

The potential of these enriched strains to trigger shrimp *V. parahaemolyticus* infection resistance under non-sterile conditions was investigated. The addition of individual strains all significantly increased the gut fullness and survival rate of the shrimp after 6 days of culture (Fig. 5a, b; Student’s *t* test* p* < 0.05). The *V. parahaemolyticus* infection resistance of the shrimp was significantly enhanced by these individual strains, with survival rates ranging from 53.3 to 66.7%, compared with 45.6% survival rate of the control group (Fig. 5c; Student’s *t* test* p* < 0.05). However, the survival rates of all individual strains were markedly lower than that of Enrich^BFS^ (Fig. 5c; Student’s *t* test *p* < 0.05). To investigate whether there was a synergistic effect of these strains on shrimp resistance, the five strains were combined to construct a SynCom^BFS^. The addition of SynCom^BFS^ had the highest gut fullness and survival rate of shrimp than that of all individual strains after 6 days of culture (Fig. 5a, b). After *V. parahaemolyticus* infection, the survival rates of SynCom^BFS^ were remarkably enhanced (Student’s *t* test *p* < 0.05) by 75.7%, compared with the control group, and were not significantly different (Student’s *t* test *p* > *0.05*) with that of the Enrich^BFS^ (Fig. 5c), indicating that the roles of SynCom^BFS^ were equivalent to that of Enrich^BFS^. Furthermore, biofilm formation was strongly increased in the SynCom^BFS^, compared with any individual strain (Table S5), further indicating that the SynCom^BFS^ could have a synergistic effect on resisting *V. parahaemolyticus* infection. To explore the potential mechanism, the digestive and immune enzyme activities were determined (Figure S13). The addition of SynCom^BFS^ and Enrich^BFS^ significantly increased the enzyme activities of AMS, CMCase, ACP, AKP, and SOD, and the addition of SynCom^BFS^ also significantly improved the enzyme activities of CAT, and total nitric oxide synthase (Figure S13; Student’s *t* test *p* < 0.05). This indicates that the high *V. parahaemolyticus* infection resistance induced by the SynCom^BFS^ might be closely associated with the improvement in the digestive and immune capacity of shrimp.

### SynCom^BFS^ addition promotes shrimp health during the entire culture process

**Fig. 6:**
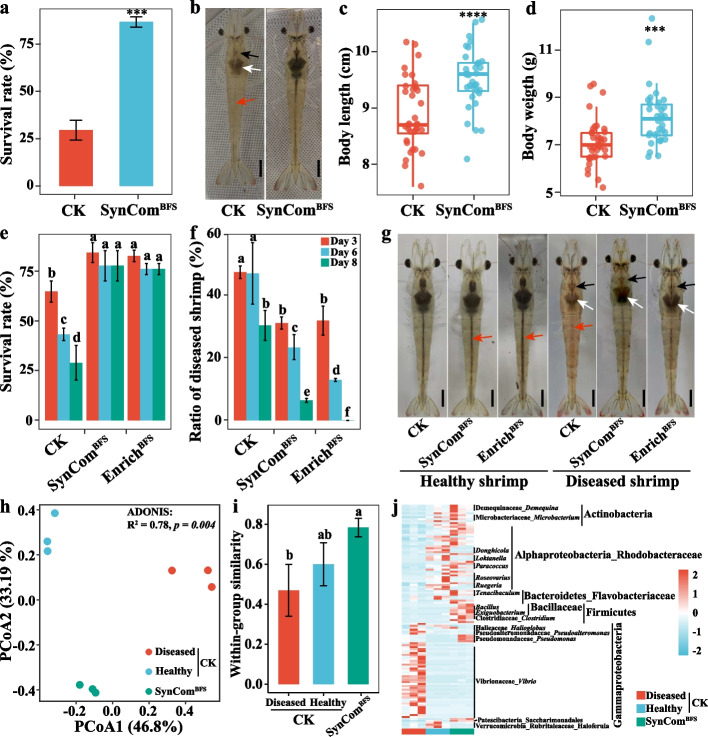
Effects of SynCom^BFS^ addition on the growth characteristics, disease recovery, and gut bacterial community of shrimp after a long time culture. **a** Survival rates, **b** phenotypic characteristics, **c** body length (*n* = 20), and **d** body weight (*n* = 20) of shrimp cultured in CK and SynCom^BFS^ addition group. The boxes represent the median and interquartile range, and whiskers range from minimum to maximum values. Different asterisks indicate a significant difference at ***p < 0.001, and ****p < 0.0001 based on Student’s *t* test. **e–g** Effects of SynCom^BFS^ and bacterial consortia on the survival rate (**e**), recovery rate (**f**), and phenotypic characteristics (**g**) of shrimp. Blank, white, and red arrows indicate the stomach, hepatopancreas, and gut of shrimp in **b** and **g**, respectively. Bar = 1 cm in **b** and **g**. **h** Principal coordinate analysis (PCoA) plot based on the Bray–Curtis dissimilarity. **i** The within-group similarities of bacterial community. Each sample had three replicates (*n* = 3), and error bars show standard deviation. Different letters indicate a significant difference at* p* < 0.05 based on Student’s *t* test. **j** The significantly discriminatory OTUs among the infected and healthy shrimp in CK and shrimp in SynCom^BFS^ addition group

The clear protection provided by SynCom^BFS^ against *V. parahaemolyticus* infection promoted the assessment of whether exogenous addition of SynCom^BFS^ could ameliorate microbiota-based protection towards shrimp disease under non-sterile conditions during the entire culture process. The results indicated that addition of SynCom^BFS^ did effectively protect the shrimp from disease. The shrimp in the control group developed the typical symptoms of *Vibrio* disease and had a low survival rate of 29.5%, while the shrimp in the SynCom^BFS^ addition group remained healthy with a high survival rate of 86.7% at 56 days of culture (Fig. 6a, b). The addition of SynCom^BFS^ also significantly improved the growth performance of shrimp, and the individual body length and body weight were enhanced by 7.0 and 15.0%, respectively, in SynCom^BFS^ addition group compared with the control (Fig. 6c, d; Student’s *t* test *p* < 0.001). Moreover, the enzyme activities of CMCase, ACP, and AKP were significantly increased in the shrimp of the SynCom^BFS^ addition group compared with the infected shrimp of the control group (Figure S14; Student’s *t* test *p* < 0.05).

To test whether the addition of SynCom^BFS^ could return the diseased shrimp back to health, control shrimp were randomly divided into three groups. The addition of SynCom^BFS^ and Enrich^BFS^ both remarkably prevented the shrimp from dying and maintained an approximate survival rate of 80% after 8 days of culture, while the survival rates of shrimp in the control group were 65.3, 43.6, and 29.2% after 3, 6, and 8 days of culture, respectively (Fig. 6e). Furthermore, the symptoms of the diseased shrimp in the SynCom^BFS^ and Enrich^BFS^ addition groups were obviously improved and even completely resolved, compared with the control group (Fig. 6f, g; Table S6), indicating that the addition of SynCom^BFS^ and Enrich^BFS^ can also cure diseased shrimp. Moreover, the addition of SynCom^BFS^ also significantly improved the survival rate of diseased shrimp postlarvae and restored their health after 4 days of culture (Table S7; Student’s *t* test *p* < 0.05).

The addition of SynCom^BFS^ strongly altered the gut bacterial community structure of shrimp based on the PCoA analysis (Fig. 6h). The within-group BC-similarities were the highest in the SynCom^BFS^ addition group, followed by the healthy shrimp and diseased shrimp in the control group (Fig. 6i), indicating that the addition of SynCom^BFS^ significantly improved the stability of the gut bacterial community. To identify the discriminatory OTUs that contributed to the divergence in bacterial community composition in the three samples, differential abundance analyses were conducted based on the DeSEq2 with OTU counts. A total of 66 OTUs and 73 OTUs were significantly enriched and depleted in the SynCom^BFS^ addition group, respectively, compared with the control group (Figure S15a-d). These significantly enriched OTUs were mainly assigned the genera of *Paracoccus*, *Ruegeria*, *Microbacterium*, *Demequina*, *Tenacibaculum*, *Bacillus*, and *Exiguobacterium*, most of which were present in the compositions of SynCom^BFS^, indicating that these taxa can colonize shrimp gut. In contrast, the remarkably depleted OTUs in the SynCom^BFS^ addition group mainly belonged to the genera of *Vibrio* (Fig. 6j), further indicating that the addition of SynCom^BFS^ can inhibit the accumulation of *Vibrio*, and thus help the shrimp to remain healthy.

## Discussion

Biofloc system (BFS) has been shown to an important culture alternative for reducing the occurrence of shrimp disease in farming practice [36]. The protective mechanisms of the BFS have been widely reported to improve shrimp vitality [37], antioxidant enzyme activities [38], and immunity gene expression [39], and to induce the phenotype switching of pathogenic bacteria [40]. Here, we showed that the gut bacterial consortium enriched in BFS shrimp efficiently resists infection by pathogenic *V. parahaemolyticus* via improving the stability and resistance of bacterial community. The significantly enriched bacterial taxa in BFS shrimp were isolated to construct a SynCom^BFS^, which significantly promoted shrimp growth, digestive and immune abilities, and disease resistance in a nature farming system. Our findings reveal the important roles of gut bacterial community and its potential mechanisms in the maintenance of the physiological health and disease resistance of shrimp.

Although a number of studies have reported that the gut bacterial community is highly associated with shrimp growth and health [10, 11], the causal relationship between them remains unknown. To confirm their causal relationship, the gut bacterial consortium from BFS shrimp was transplanted to the guts of WES shrimp. The survival rate of WES shrimp that received Enrich^BFS^ was significantly enhanced and equivalent to that of BFS shrimp (Fig. 1b, c), confirming that the protective effect of the BFS against *Vibrio* infection was at least partially caused by the BFS-induced shrimp gut bacterial community. Furthermore, the addition of Enrich^BFS^ into the culture system also significantly improved the *Vibrio* infection resistance of shrimp postlarvae (Fig. 4d–f), further verifying that the bacterial community induced by the BFS protected the shrimp from *Vibrio* infection.

The stability of the host microbiome is considered key for host health, because it guarantees that beneficial symbionts and their related functions are maintained over time [41]. Previous studies have indicated that the stability of the gut bacterial community is significantly increased in BFS shrimp [8, 13], and promoted us to construct connections between improved resistance and bacterial community stability. In this study, the BFS shrimp with higher resistance against *Vibrio* infection were characterized by a more stable gut bacterial community, including lower within-group variation, a more complex co-occurrence network, and higher robustness (Figs. 2a, b, and S5). The transplantation of Enrich^BFS^ into WES and BFS shrimp significantly enhanced the within-group BC-similarity of the gut bacterial community (Fig. 3b). Meanwhile, the addition of Enrich^BFS^ into the culture system remarkably increased the within-group BC-similarity, bacterial network complexity and stability, and robustness (Figs. 4g, h, and S9). The bacterial community stability parameters were significantly and positively correlated with survival rate of shrimp after pathogen infection (Figs. 2d, 3j, and 4g). Overall, these findings demonstrate that the stability of gut bacterial community might be an important factor behind higher pathogen infection resistance in the BFS. This was consistent with that stability of microbial community driven by resistance and resilience protected the host from dysbiosis-related diseases and various environment stresses [42, 43].

Previous studies have shown that several taxa from the families of Rhodobacteraceae, Flavobacteriaceae and Microbacteriaceae were significantly enriched in the BFS [8, 44] or healthy shrimp [11]. These enriched taxa were closely associated with the shrimp growth performance and health, and were considered potential biocontrol species for the protection of shrimp from pathogen infection [10, 45]. Our data indicated that the relative abundances of *Ruegeria*, *Paracoccus*, *Microbacterium*, *Demequina*, and *Tenacibaculum* were significantly enriched in the BFS (Figure S11b). However, it is a major challenge to reveal the causal relationships, as opposed to just associations, between these enriched taxa and host phenotype in shrimp. FMT has been widely applied to study the physiological significance of the gut microbiome in a large number of animals and in humans [13, 46]. The most common way to collect fecal microbiota is by extracting a suspension of fecal samples [46]. This not only requires a large number of fecal samples, but also is difficult for the application of vast propagation in practical shrimp farming systems. Recently, some researchers have attempted to obtain the microbial consortia from the rhizosphere and endosphere by enrichment culture, and these consortia have revealed an excellent ability in promoting plant growth under salt stress [22] or enhancing resistance against pathogen infection [47]. In the present study, a BFS shrimp gut bacterial consortium (Enrich^BFS^) was first obtained via enrichment culture in R2A medium using the gut fecal extraction as the inoculum. Although the total relative abundances of potential biocontrol taxa, such as *Ruegeria*, *Paracoccus*, *Microbacterium*, *Demequina*, and *Tenacibaculum* in Enrich^BFS^, were less than 10%, the transplantation of Enrich^BFS^ into both WES and BFS shrimp significantly enhanced their *Vibrio* infection resistance (Fig. 2b, c). This was consistent with that the probiotic roles of a microbial consortium in host health are not dependent on the abundance of each strain, but rather the functional profiles of these strains [48, 49].

The colonization of the gut by microbial communities is a necessary step for the control of various diseases in human and animals [21, 50]. The present data showed that both transplantation and addition of Enrich^BFS^ significantly increased the relative abundances of *Ruegeria*, *Paracoccus*, *Microbacterium* and *Tenacibaculum* in shrimp (Fig. 3e), indicating that the improved *Vibrio* infection resistance by Enrich^BFS^ might benefit from the colonization of these potential biocontrol taxa in shrimp. Most notably, although the relative abundance of *Vibrio* was as high as 90% in Enrich^BFS^ (Figures S6a and S12a), it still played an important role in improving shrimp growth and *Vibrio* infection resistance (Figs. 2b and 4d–f). It is speculated that the existence of high diversity taxa in the Enrich^BFS^ might extremely hinder the colonization of *Vibrio* in shrimp guts because the relative abundance of *Vibrio* decreased after both transplantation and addition of Enrich^BFS^ (Figs. 3e and S9d). This was consistent with that certain taxa from Rhodobacteraceae, Microbacteriaceae and Flavobacteriaceae can efficiently resist the colonization of *Vibrio* in shrimp guts by improving the gut bacterial community stability or secreting antibacterial substances [9, 51, 52]. Therefore, these species enriched in the bacterial consortium may be closely associated with the healthy growth and disease resistance of shrimp.

Although SynCom construction is key for the functional confirmation and practical application of microbes, how to design the strain combination and measuring their compatibility and ecological stability are laborious and time-consuming work [53]. Here, we proposed an innovative approach to integrate enrichment culture and SynCom for verifying the ability of a BFS-enriched bacterial community to resist *Vibrio* infection. First, a bacterial consortium with high *Vibrio* infection resistance were obtained by enriching the BFS shrimp gut bacterial community in R2A media. By determining the microbial community composition of the bacterial consortium, five highly enriched strains including two from Rhodobacteraceae, two from Actinobacteria, and one from Bacteroidetes, were isolated to construct SynCom^BFS^ (Tables S2 and S3), which significantly improved the resistance of shrimp against *Vibrio* infection (Fig. 5c). This approach not only guided the screening of bacterial species for building appropriate combinations, but also allowed the direct isolation of these strains from the enrichment culture, thus helping to improve the efficiency of assembling disease-resistant microbial communities [22, 49].

Of note, the roles of SynCom^BFS^ in improving shrimp fitness and *Vibrio* infection resistance were equivalent to the addition of Enrich^BFS^ (Fig. 5c). Previous studies indicated that the BFS improved shrimp health by enhancing the digestive enzyme activities and immune response of shrimp [38, 54]. Therefore, we studied the effects of Enrich^BFS^ and SynCom^BFS^ addition on the digestive and immune enzyme activities of the shrimp. The addition of Enrich^BFS^ and SynCom^BFS^ significantly increased the enzyme activities of CMCase, ACK, APK, CAT, and SOD (Figures S8 and S13), which were also significantly improved in BFS shrimp (Figure S3). This indicated that the bacterial community induced by the BFS could improve the digestive capacity and antioxidant status of shrimp. It has been reported that some beneficial gut bacteria can activate innate immunity during interactions with the host [55]. Antioxidant enzymes (such as CTA and SOD) play important roles in the maintenance of immune homeostasis when the organism encounters pathogen infection [56]. ACK and APK can induce autophagy, a vital innate immune pathway known for its role in barrier function, inflammation, and antimicrobial function, and increased their activities could positively regulate antimicrobial proteins such as lysozymes that control pathogen invasion [57, 58].

The common purpose of constructing SynComs is to maintain traits that are beneficial to host health in the field [59]. Previous studies have attempted to combat disease with SynComs in sterile conditions [60]. However, whether a SynCom can provide protection against pathogen invasion to host in the existence of a natural or local microbiome under field conditions is mostly unknown. Here, SynCom^BFS^ addition experiments were performed in an open culture system. The addition of SynCom^BFS^ not only improved the growth performance of shrimp, but also prevented the occurrence of disease (Fig. 6a–d). Of note, the addition of SynCom^BFS^ also alleviated the symptoms of diseased shrimp and even returned the diseased shrimp to health (Fig. 6e–g). These results support the notion that SynCom^BFS^ as a community is required for enhancing disease resistance in shrimp. Moreover, the addition of SynCom^BFS^ significantly altered the gut bacterial community structure of shrimp with lower within-group variation (Fig. 6h, i), indicating that SynCom^BFS^ addition might suppress disease occurrence by increasing the stability of the gut bacterial community. The strains were able to adapt rapidly to their original habitat, and thus colonize effectively [34]. Here, the addition of SynCom^BFS^ significantly increased the relative abundances of *Demequina*, *Microbacterium*, *Paracoccus*, *Ruegeria*, and *Tenacibaculum* (Fig. 6j), indicating that the members of SynCom^BFS^ effectively colonize the shrimp gut. The combination of strains from SynCom^BFS^ also showed a synergistic effect in biofilm formation (Figure S15), which might potentially trigger bacterial colonization and subsequent host disease resistance [61, 62].

## Conclusions

**Fig. 7:**
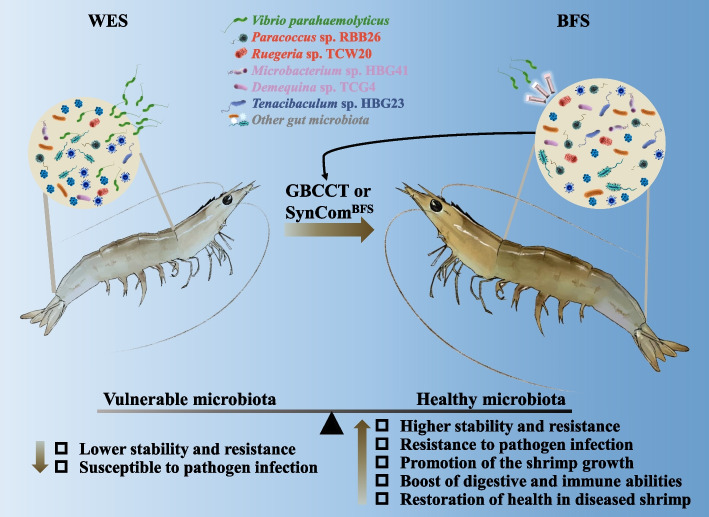
The potential mechanisms of bacterial community enriched in the BFS against *Vibrio parahaemolyticus* infection. The BFS induced a healthy microbiota that show the following characteristics: higher stability and resistance, resistance to pathogen infection, promotion of the shrimp growth, boost of digestive and immune abilities, and restoration of health in diseased shrimp

Overall, these findings reveal the microecology mechanism of disease resistance mediated by a BFS shrimp gut bacterial community (Fig. 7). The BFS could induce the assemblage of a beneficial bacterial consortium, which improves the stability and resistance of gut bacterial community, and digestive and immune abilities of shrimp to promote the growth and disease resistance of host. Our study confirms that the BFS shapes a stable and resistant gut bacterial community to protect shrimp against pathogenic bacteria infection. The introduction of SynCom to enhance the stability of gut bacterial community could be a potential avenue of intervention for the improvement of fitness and disease resistance in shrimp.

### Supplementary Information

**Additional file 1:** **Figure S1.** Experimental scheme for comprehensively exploring the disease resistance of shrimp gut bacterial consortium induced by a biofloc system. **Figure S2.** The growth characteristics and *Vibrio* infection resistance of shrimp cultured in the WES and BFS. **Figure S3.** The digestive and immune enzyme activities in the hepatopancreas of shrimp cultured in the WES and BFS for 21 d. **Figure S4.** The α-diversity characteristics, structures, and compositions of the bacterial communities in the WES and BFS after 21 d of culture. **Figure S5.** The bacterial co-occurrence network characteristics of the WES and BFS after 21 d of culture. **Figure S6.** The compositions of gut bacterial consortia enriched from WES and BFS shrimp. **Figure S7.** The experimental systems for shrimp postlarvae culture and *Vibrio* infection, and the formula for calculating the gut fullness. **Figure S8.** Effects of gut bacterial consortia addition on digestive and immune enzyme activities of shrimp postlarvae after 6 d of culture. **Figure S9.** The α-diversity characteristics, structures, and compositions of the bacterial community in CK, Enrich^WES^ and Enrich^BFS^. **Figure S10.** The bacterial co-occurrence network characteristics in CK, Enrich^WES^ and Enrich^BFS^. **Figure S11.** The survival rates and bacterial community compositions of WES and BFS shrimp in the first and second culture experiments. **Figure S12.** The compositions of bacterial consortia enriched from the first and second culture shrimp, and survival rate of shrimp after cross-transplantation or addition experiments. **Figure S13.** The effects of SynCom^BFS^ and Enrich^BFS^ addition on the digestive and immune enzyme activities of shrimp postlarvae after 6 d of culture. **Figure S14.** The digestive and immune enzyme activities of shrimp cultured in CK and SynCom^BFS^ addition groups after 56 d of culture. **Figure S15.** Effects of SynCom^BFS^ addition on the gut bacterial community compositions of shrimp after 56 d of culture.

**Additional file 2:** **Table S1.** The information of shrimp used in this study and their corresponding results. **Table S2.** The assigned genus, and relative abundance of the significantly enriched OTUs in BFS and enrichment culture from BFS shrimp. **Table S3.** The informations of isolated strains from shrimp culture system and baterial consortium from BFS shrimp and their identification based on the 16S rRNA gene sequence. **Table S4.** The top-hit strains of five significantly enriched OTUs and their identification based on the genome sequence. **Table S5.** The potential probiotic functions of five selected strains. **Table S6.** The recovery role of synthetic bacterial community on diseased adult shrimp in control group. **Table S7.** The recovery role of synthetic bacterial community on diseased juvenile shrimp in control group.

## Data Availability

The datasets supporting the conclusions of this article are available in the NCBI Sequence Read Archive database with the unique identifier PRJNA953753 (https://www.ncbi.nlm.nih.gov/sra/?term=PRJNA953753) for amplicon sequencing.
