# Supplementary material for: Gut bacterial consortium enriched in a biofloc system protects shrimp against Vibrio parahaemolyticus infection
Source: Microbiome. 2023 Oct 19;11:230. doi: 10.1186/s40168-023-01663-2 (PMC10585862; doi:10.1186/s40168-023-01663-2)
Supplement: Supplementary file 2 — Additional file 1: Figure S1. Experimental scheme for comprehensively exploring the disease resistance of shrimp gut bacterial consortium induced by a biofloc system. Figure S2. The growth characteristics and Vibrio infection resistance of shrimp cultured in the WES and BFS. Figure S3. The digestive and immune enzyme activities in the hepatopancreas of shrimp cultured in the WES and BFS for 21 d. Figure S4. The α-diversity characteristics, structures, and compositions of the bacterial communities in the WES and BFS after 21 d of culture. Figure S5. The bacterial co-occurrence network characteristics of the WES and BFS after 21 d of culture. Figure S6. The compositions of gut bacterial consortia enriched from WES and BFS shrimp. Figure S7. The experimental systems for shrimp postlarvae culture and Vibrio infection, and the formula for calculating the gut fullness. Figure S8. Effects of gut bacterial consortia addition on digestive and immune enzyme activities of shrimp postlarvae after 6 d of culture. Figure S9. The α-diversity characteristics, structures, and compositions of the bacterial community in CK, EnrichWES and EnrichBFS. Figure S10. The bacterial co-occurrence network characteristics in CK, EnrichWES and EnrichBFS. Figure S11. The survival rates and bacterial community compositions of WES and BFS shrimp in the first and second culture experiments. Figure S12. The compositions of bacterial consortia enriched from the first and second culture shrimp, and survival rate of shrimp after cross-transplantation or addition experiments. Figure S13. The effects of SynComBFS and EnrichBFS addition on the digestive and immune enzyme activities of shrimp postlarvae after 6 d of culture. Figure S14. The digestive and immune enzyme activities of shrimp cultured in CK and SynComBFS addition groups after 56 d of culture. Figure S15. Effects of SynComBFS addition on the gut bacterial community compositions of shrimp after 56 d of culture. [file 40168_2023_1663_MOESM1_ESM.pdf]

Gut bacterial consortium enriched in a biofloc system protects shrimp against *Vibrio parahaemolyticus* infection

Haipeng Guo[1,2#]*, Xuezhi Fu[1,2#], Jikun He[1,2], Ruoyu Wang[1,2], Mengchen Yan[1,2], Jing Wang[1,2], Pengsheng Dong[1,2], Lei Huang[1,2], Demin Zhang[1,2]*

[1] State key laboratory for managing biotic and chemical threats to the quality and safety of agro-products, Ningbo University, Ningbo 315211, China

[2] School of Marine Sciences, Ningbo University, Ningbo, 315211, China

[#]These authors have contributed equally to this work.

Corresponding authors:

*E-mail: guohaipeng@nbu.edu.cn (Haipeng Guo); zhangdemin@nbu.edu.cn (Demin Zhang)

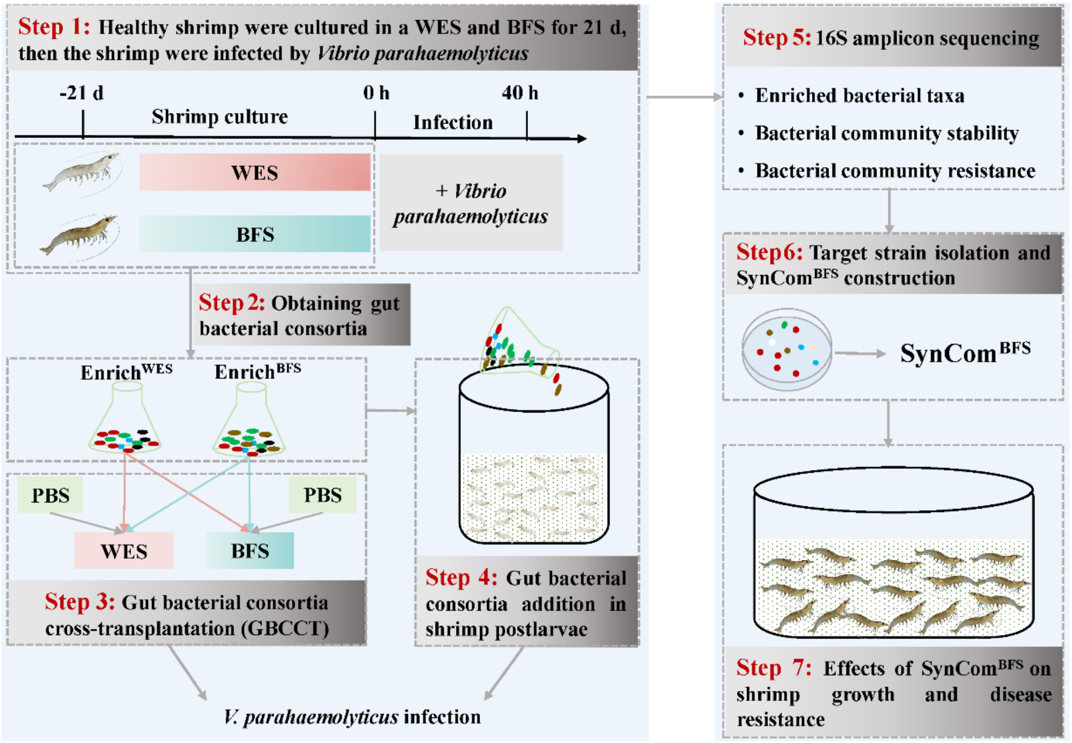

Figure S1 Experimental scheme for comprehensively exploring the disease resistance of shrimp gut bacterial consortium induced by a biofloc system. A total of seven steps were involved in this study. The higher resistance of pathogenic *Vibrio parahaemolyticus* in BFS shrimp was confirmed in step 1. In the steps 2-4, the gut bacterial consortium were obtained from WES and BFS shrimp, respectively, to confirm the causal relationship between shrimp disease resistance and the gut bacterial community via cross-transplantation and addition experiments. The gut bacterial communities of shrimp cultured in the WES and BFS after cross-transplantation or addition of bacterial consortium were characterized, and five enriched bacterial taxa were identified in step 5. In step 6, the corresponding strains of five enriched bacterial taxa were isolated from shrimp gut and bacterial consortium to construct a SynCom^{BFS}. In the last step, the promotive roles and potential mechanisms of SynCom^{BFS} on shrimp growth and disease resistances were revealed by the culture experiments. WES, Water exchange system; BFS, Biofloc system.

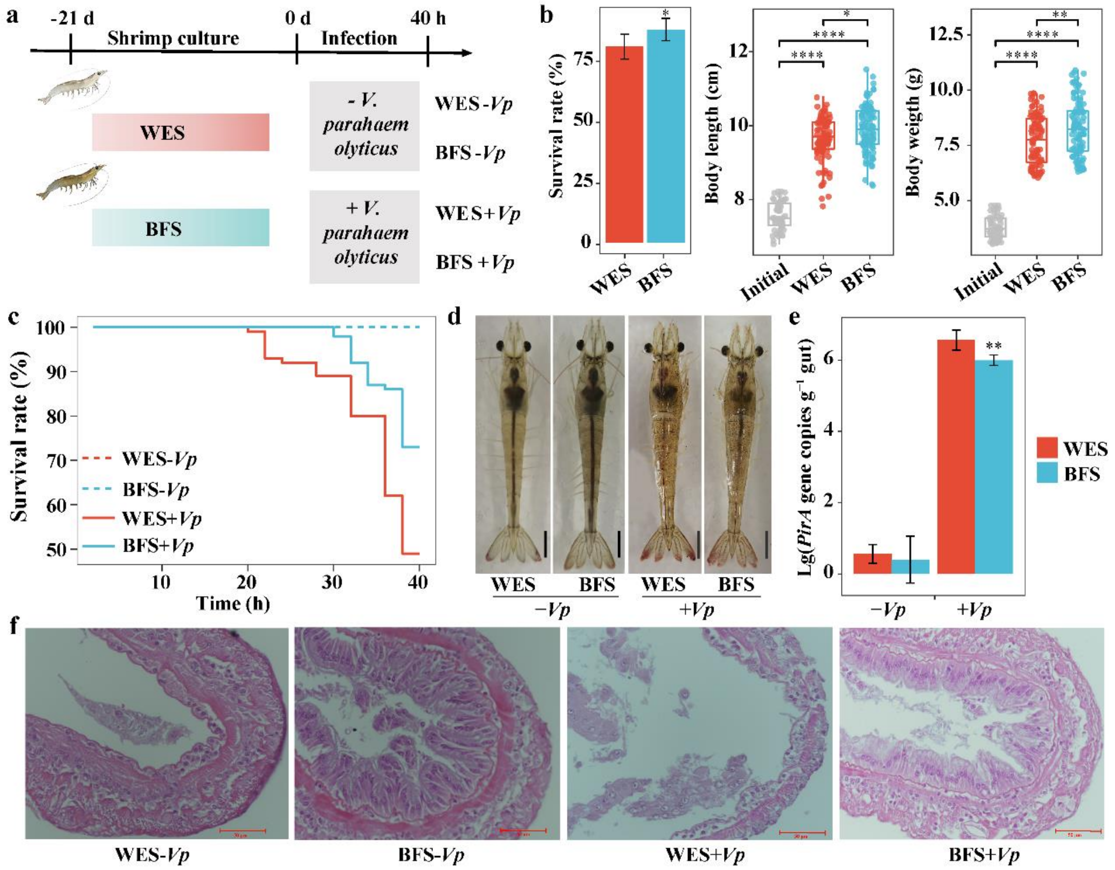

Figure S2 The growth characteristics and *Vibrio* infection resistance of shrimp cultured in the WES and BFS. (a) Schematic diagram of the experimental procedure. The healthy juvenile shrimp were first cultured in two different culture systems for 21 d, and then they were infected by a pathogenic *Vibrio parahaemolyticus*. (b) The differences in survival rates, body length (n=20), and body weight (n=20) of shrimp. The survival rate (c), phenotypic characteristic (d), *PirA* gene copy numbers (e), and histological sections of shrimp midgut (f) after *Vibrio parahaemolyticus* infection. Different asterisks indicate a significant difference at *$p < 0.05$, **$p < 0.01$, and ****$p < 0.0001$ based on Student's t-test. Each sample had five replicates (n=5), and error bars show standard deviation. Bar = 1 cm in (d), and = 50 μm in (f). WES, Water exchange system; BFS, Biofloc system; -*Vp*, non-*Vibrio* infection; +*Vp*, *Vibrio* infection.

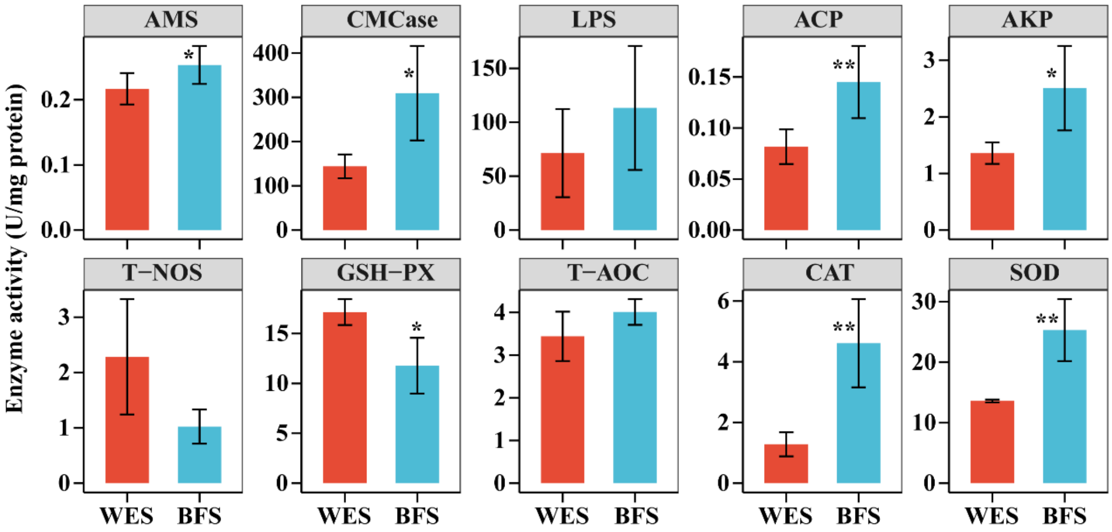

Figure S3 The digestive and immune enzyme activities in the hepatopancreas of shrimp cultured in the WES and BFS for 21 d. Each sample had five replicates (n=5), and error bars show standard deviation. Different asterisks indicate a significant difference at *$p < 0.05$ and **$p < 0.01$ based on Student's t-test. WES, Water exchange system; BFS, Biofloc system; AMS, Amylase; CMCase, Carboxymethyl cellulase; LPS, Lipase; ACP, Acid phosphatase; AKP, Alkaline phosphatase; T-NOS, Total nitric oxide synthase; GSH-PX, Glutathione peroxidase; T-AOC, Total anti-oxidative capacity; CAT, Catalase; SOD, Superoxide dismutase.

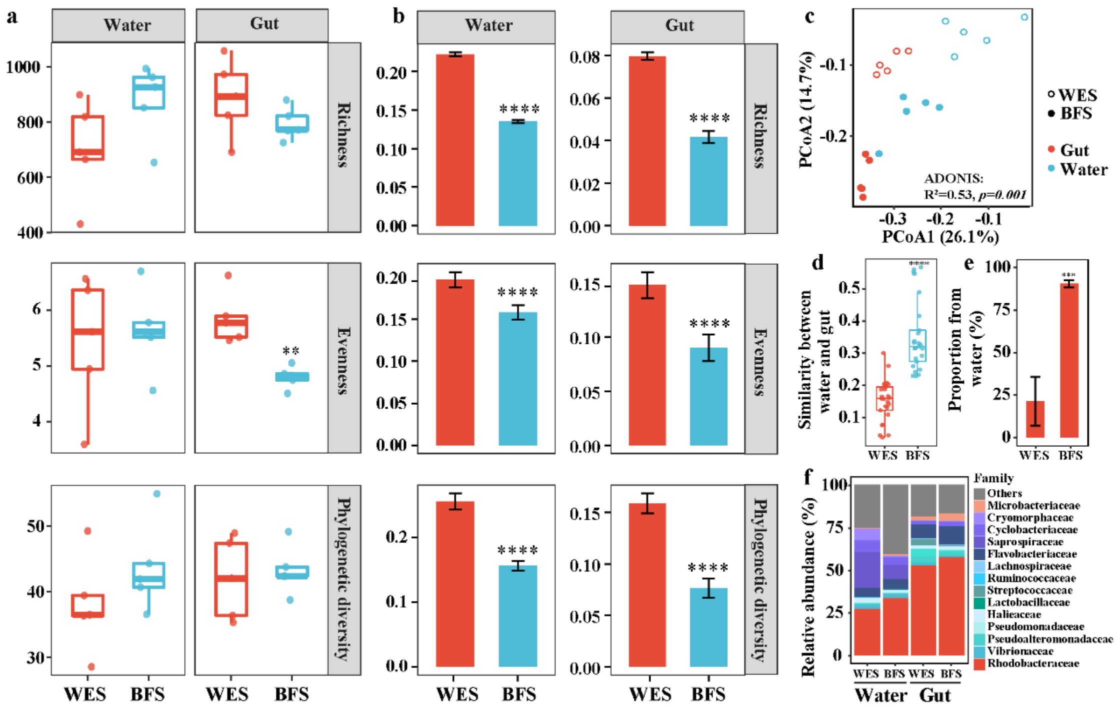

Figure S4 The α-diversity characteristics, structures, and compositions of the bacterial communities in the WES and BFS after 21 d of culture. (a) The α-diversity indexes, including richness, evenness, and phylogenetic diversity in the WES and BFS. (b) The variable coefficient of α-diversity indexes. (c) Principal coordinate analysis (PCoA) plot based on the Bray-Curtis dissimilarity. (d) The pairwise similarities of bacterial community. (e) The percentage contributions of bacterial sources for gut samples in each culture system based on SourceTracker analysis. (f) The relative abundances of key taxa at the family level in the WES and BFS. The boxes represent the median and interquartile range, and whiskers range from minimum to maximum values. Each sample had five replicates (n=5), and the error bars show standard deviation. Different asterisks indicate a significant difference at ***$p < 0.01$* and ****$p < 0.0001$* based on Student's t-test. WES, Water exchange system; BFS, Biofloc system.

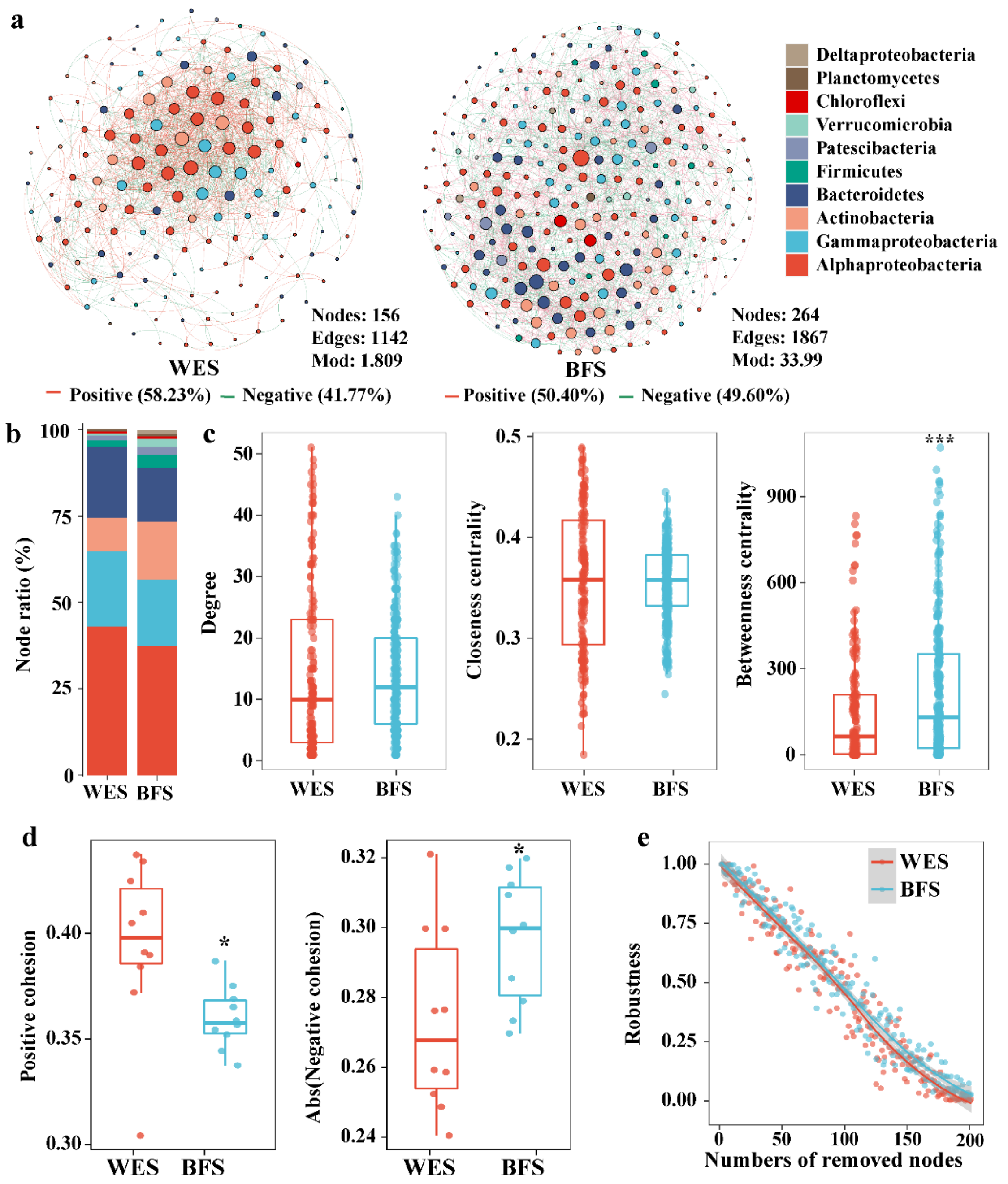

Figure S5 The bacterial co-occurrence network characteristics of the WES and BFS after 21 d of culture. (a) The co-occurrence networks of bacterial communities in the WES and BFS. (b) The node ratio of co-occurrence networks. (c) The degree, closeness centrality and betweenness centrality of co-occurrence networks. (d) The values of positive cohesion, and absolute value of negative cohesion. (e) Changes in robustness (natural connectivity) of bacterial networks. The boxes represent the median and interquartile range, and whiskers range from minimum to maximum values. Each sample had five replicates (n=5), and error bars show standard deviation. Different asterisks indicate a significant difference at *$p < 0.05$*, ***$p < 0.001$*, and ****$p < 0.0001$* based on Student's t-test. WES, Water exchange system; BFS, Biofloc system.

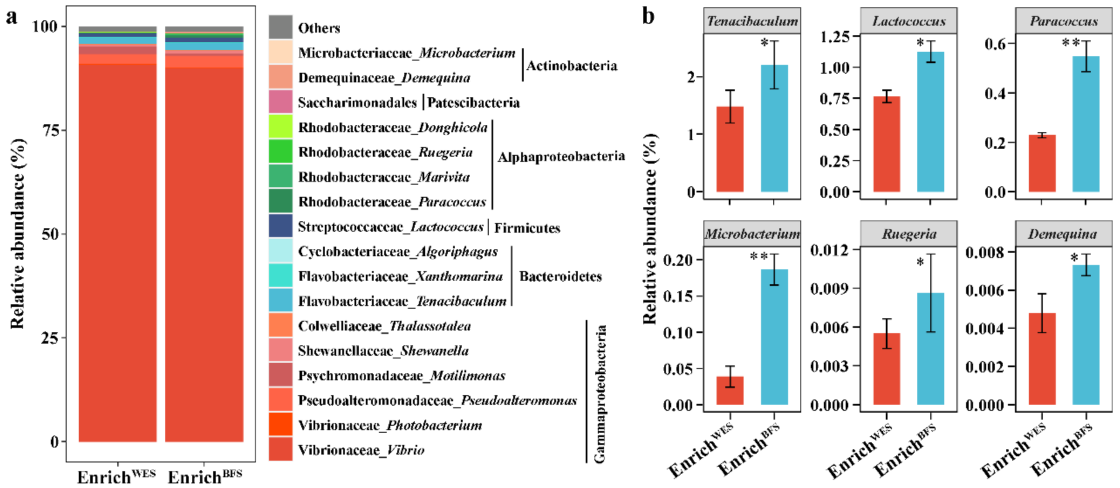

Figure S6 The compositions of gut bacterial consortia enriched from WES and BFS shrimp. (a) Relative abundances of bacterial community. (b) Key OTUs showing significant differences. Each sample had three replicates (n=3), and error bars show standard deviation. Different asterisks indicate a significant difference at $*p < 0.05$ and $**p < 0.01$ based on Student's t-test. Enrich^{WES}, Gut bacterial consortium obtained from WES shrimp; Enrich^{BFS}, Gut bacterial consortium obtained from BFS shrimp; OTU, Operational taxonomic units.

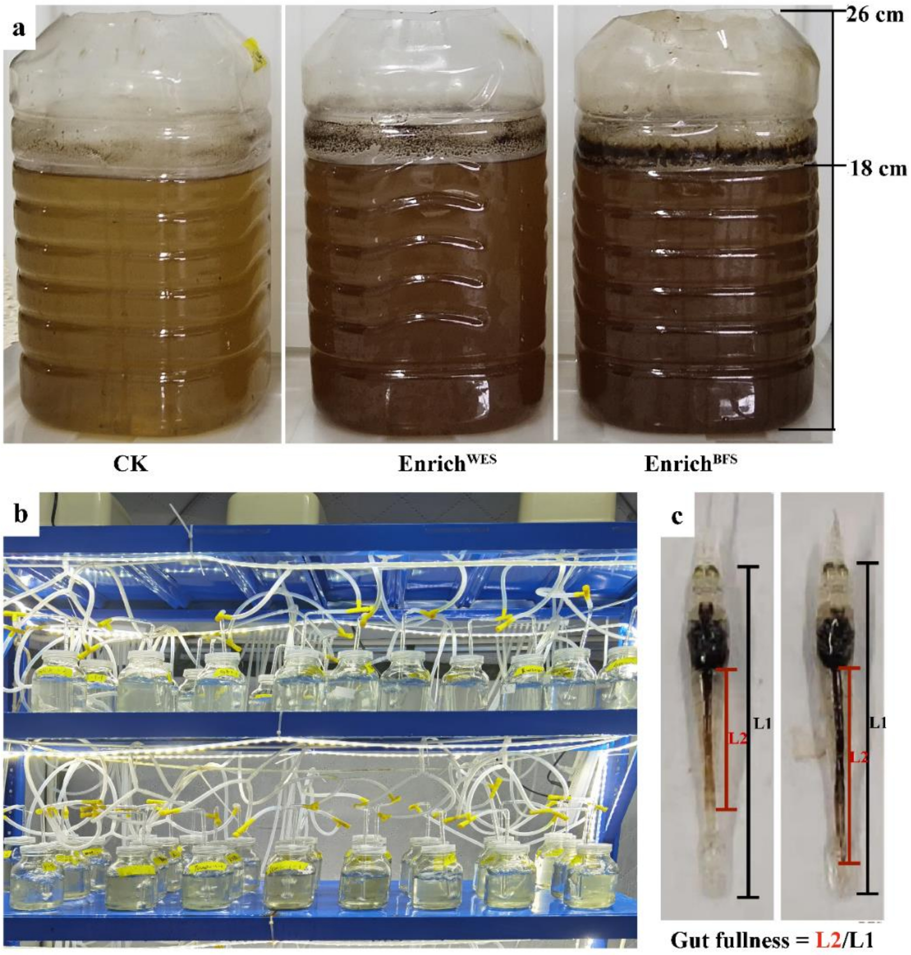

Figure S7 The experimental systems for shrimp postlarvae culture and *Vibrio* infection, and the formula for calculating the gut fullness. (a) The 5-L culture system for postlarvae and the rearing water color after 6 d of culture. (b) The 650-mL pathogen infection system for postlarvae. (c) The gut fullness is obtained by calculating the ratio of gut length to body length. CK, Control; Enrich^{WES}, Addition of bacterial consortium enriched from WES shrimp; Enrich^{BFS}, Addition of bacterial consortium enriched from BFS shrimp.

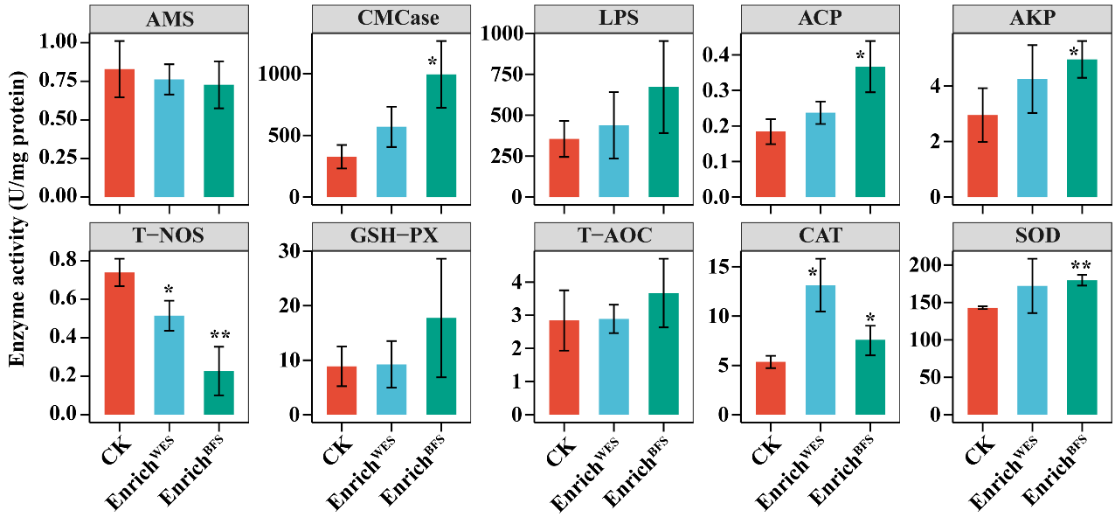

Figure S8 Effects of gut bacterial consortia addition on digestive and immune enzyme activities of shrimp postlarvae after 6 d of culture. Each sample had five replicates (n=5), and error bars show standard deviation. Different asterisks indicate a significant difference at $*p < 0.05$ and $**p < 0.01$ based on Student's t-test. CK, Control; Enrich^{WES}, Addition of the gut bacterial consortium enriched from WES shrimp; Enrich^{BFS}, Addition of the gut bacterial consortium enriched from BFS shrimp; AMS, Amylase; CMCase, Carboxymethyl cellulase; LPS, Lipase; ACP, Acid phosphatase; AKP, Alkaline phosphatase; T-NOS, Total nitric oxide synthase; GSH-PX, Glutathione peroxidase; T-AOC, Total anti-oxidative capacity; CAT, Catalase; SOD, Superoxide dismutase.

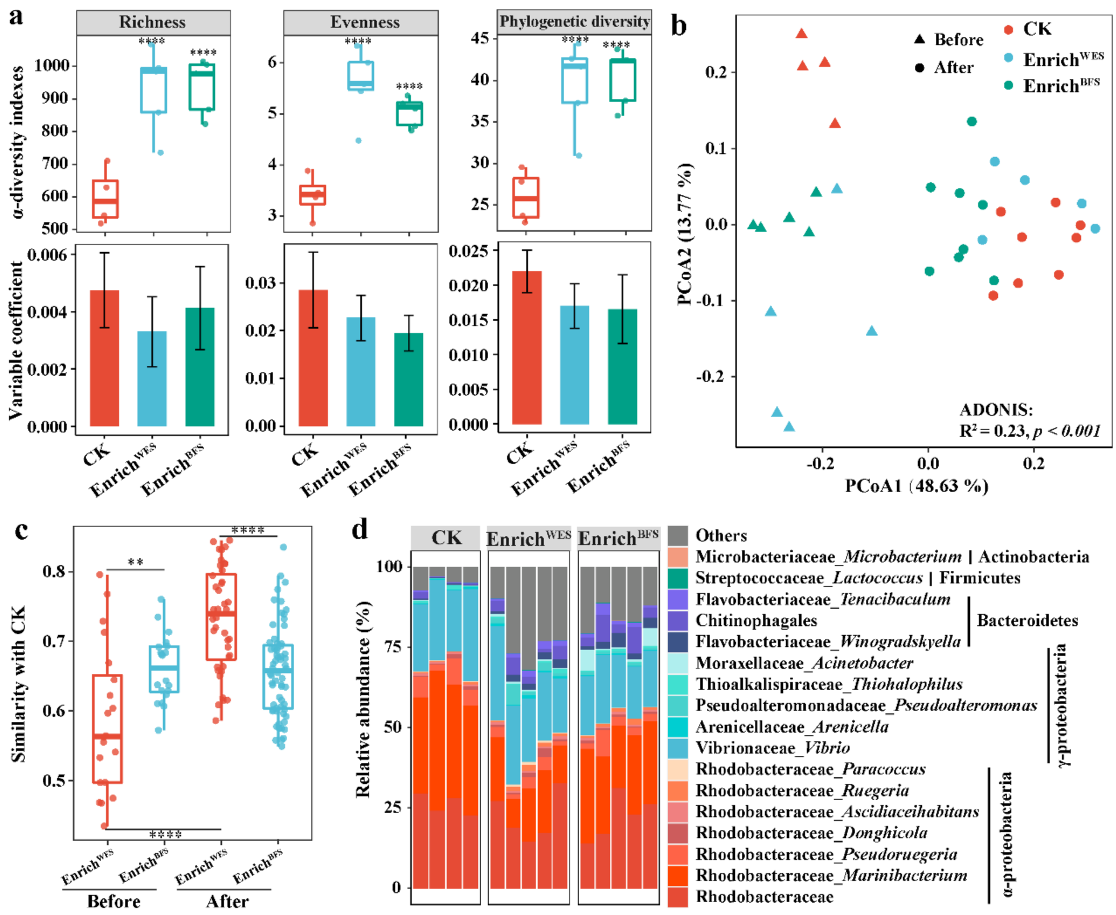

Figure S9 The α-diversity characteristics, structures, and compositions of the bacterial community in CK, Enrich^{WES} and Enrich^{BFS}. (a) The α-diversity indexes, including richness, evenness and phylogenetic diversity, and the variable coefficient of α-diversity indexes. (b) Principal coordinate analysis (PCoA) plot based on the Bray-Curtis dissimilarity. (c) The bacterial community similarities between the control and bacterial consortia addition groups before and after pathogen infection. The boxes represent the median and interquartile range, whiskers range from minimum to maximum values, and error bars show standard deviation. Different asterisks indicate a significant difference at ****$p < 0.0001$ based on Student's t-test. (d) The relative abundances of key bacterial taxa at the genus level. Each sample had four replicates in CK (n=4) and five replicates in Enrich^{WES} and Enrich^{BFS} (n=5). CK, Control; Enrich^{WES}, Addition of the gut bacterial consortium enriched from WES shrimp; Enrich^{BFS}, Addition of the bacterial consortium enriched from BFS shrimp.

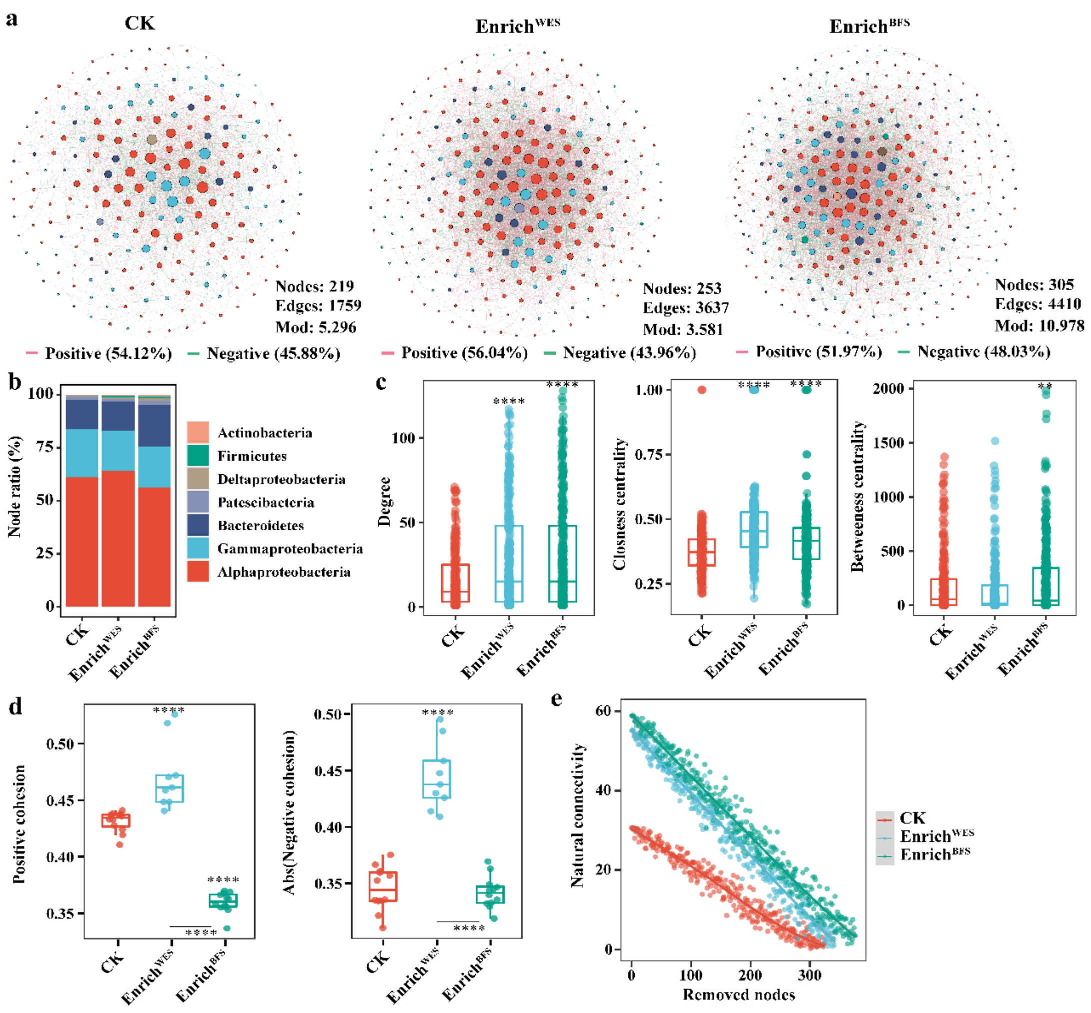

Figure S10 The bacterial co-occurrence network characteristics in CK, Enrich^{WES} and Enrich^{BFS}. (a) The co-occurrence networks of bacterial communities. (b) The node ratio of co-occurrence networks. (c) The degree, closeness centrality and betweenness centrality of co-occurrence networks. (d) The values of positive cohesion, and absolute value of negative cohesion. (e) The natural connectivity of bacterial networks. The boxes represent the median and interquartile range, whiskers range from minimum to maximum values. Each sample had five replicates (n=5). Different asterisks indicate a significant difference at **$p < 0.01$, ***$p < 0.001$ and ****$p < 0.0001$ based on Student's t-test. CK, Control; Enrich^{WES}, Addition of the gut bacterial consortium enriched from WES shrimp; Enrich^{BFS}, Addition of the gut bacterial consortium enriched from BFS shrimp.

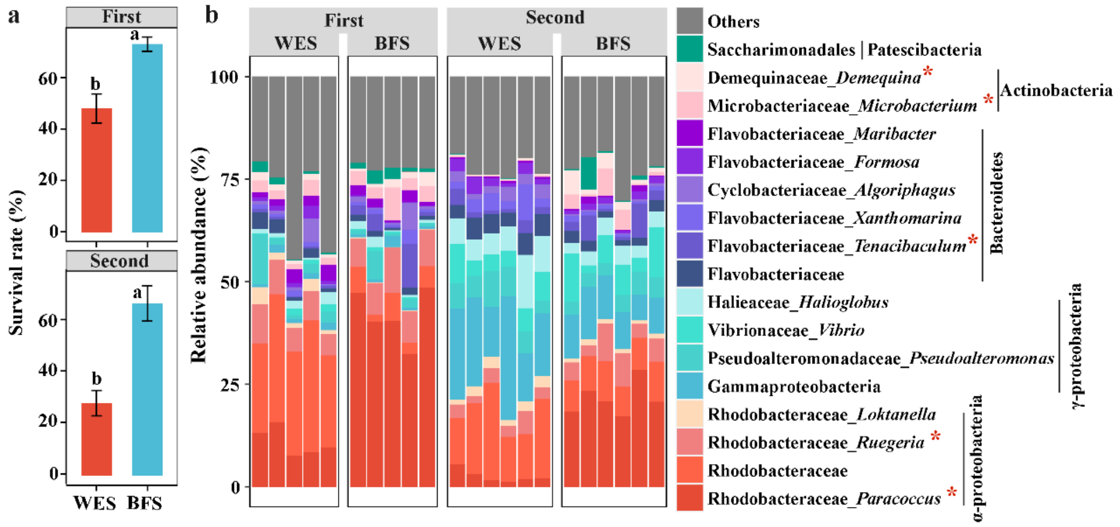

Figure S11 The survival rates and bacterial community compositions of WES and BFS shrimp in the first and second culture experiments. (a) The survival rates after 21 d of culture. Each sample had five replicates (n=5) in the first culture and six replicates (n=6) in the second culture experiment, and error bars show standard deviation. Different letters indicate a significant difference at $*p < 0.05$ based on Student's t-test. (b) The relative abundances of key taxa at the genus level. The red asterisks indicate that the bacterial taxa were significantly different at genus level between WES and BFS in two separate experiments (Student's t-test $p < 0.05$). WES, Water exchange system; BFS, Biofloc system.

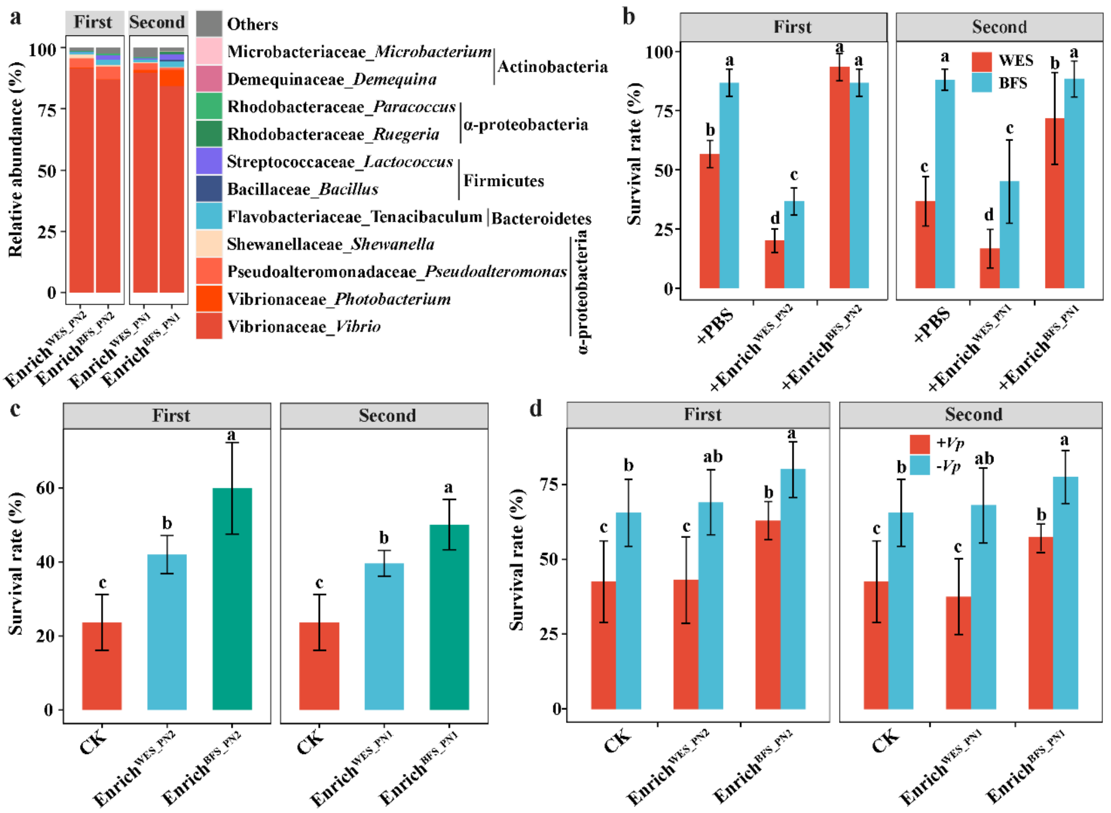

Figure S12 The compositions of bacterial consortia enriched from the first and second culture shrimp, and survival rate of shrimp after cross-transplantation or addition experiments. (a) The relative abundances of key taxa at the genus level in the bacterial consortia. The shrimp survival rates after cross-transplantation of bacterial consortia (b), after adding the bacterial consortia (c) and after *Vibrio* infection (d). Each sample had three replicates (n=3) in the first and second culture experiment, and error bars show standard deviation. Different letters indicate a significant difference at $*p < 0.05$ based on Student's t-test. CK, Control; Enrich^{WES}, addition of the bacterial consortia enriched from WES shrimp; Enrich^{BFS}, addition of the bacterial consortia enriched from BFS shrimp; PN1, Passage number is one; PN2, Passage number is two; -*Vp*, non-*Vibrio* infection; +*Vp*, *Vibrio* infection.

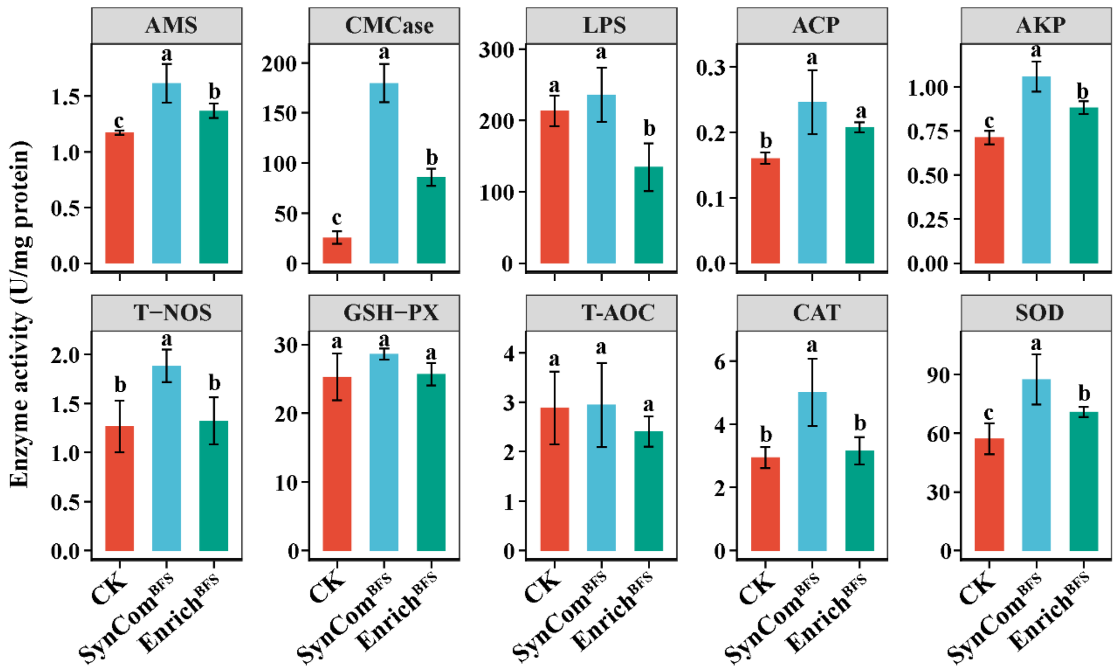

Figure S13 The effects of SynCom^{BFS} and Enrich^{BFS} addition on the digestive and immune enzyme activities of shrimp postlarvae after 6 d of culture. Each sample had five replicates (n=5), and error bars show standard deviation. Different letters indicate a significant difference at *$p < 0.05$* based on Student's t-test. AMS, Amylase; CMCase, Carboxymethyl cellulase; LPS, Lipase; ACP, Acid phosphatase; AKP, Alkaline phosphatase; T-NOS, Total nitric oxide synthase; GSH-PX, Glutathione peroxidase; T-AOC, Total anti-oxidative capacity; CAT, Catalase; SOD, Superoxide dismutase; Enrich^{BFS}, Gut bacterial consortium enriched from BFS shrimp.

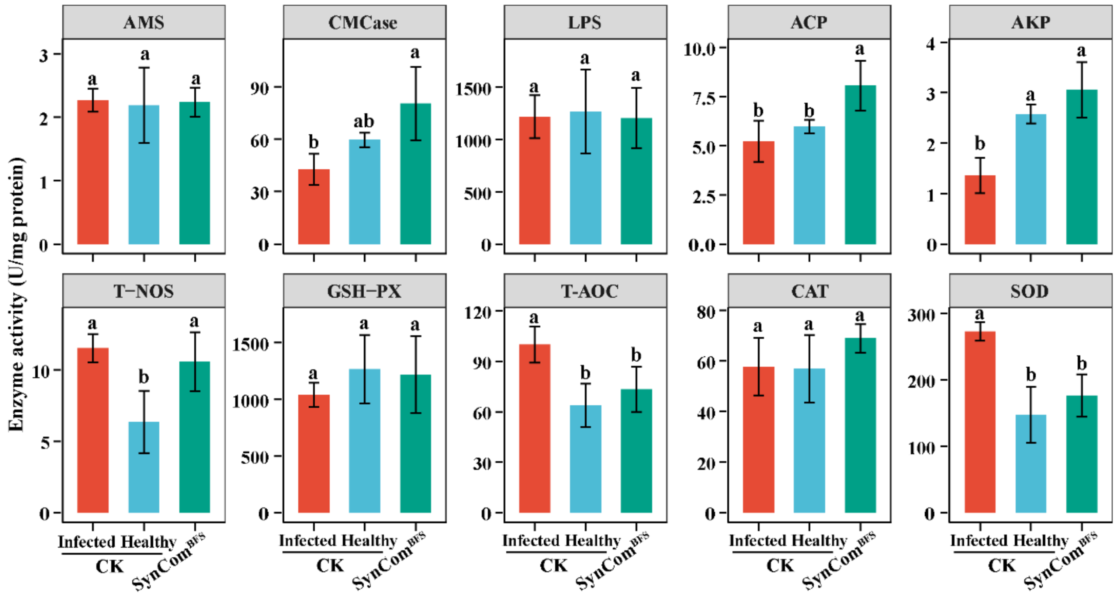

Figure S14 The digestive and immune enzyme activities of shrimp cultured in CK and SynCom^{BFS} addition groups after 56 d of culture. Each sample had three replicates (n=3), and error bars show standard deviation. Different letters indicate a significant difference at **p* < 0.05* based on Student's t-test. AMS, Amylase; CMCase, Carboxymethyl cellulase; LPS, Lipase; ACP, Acid phosphatase; AKP, Alkaline phosphatase; T-NOS, Total nitric oxide synthase; GSH-PX, Glutathione peroxidase; T-AOC, Total anti-oxidative capacity; CAT, Catalase; SOD, Superoxide dismutase.

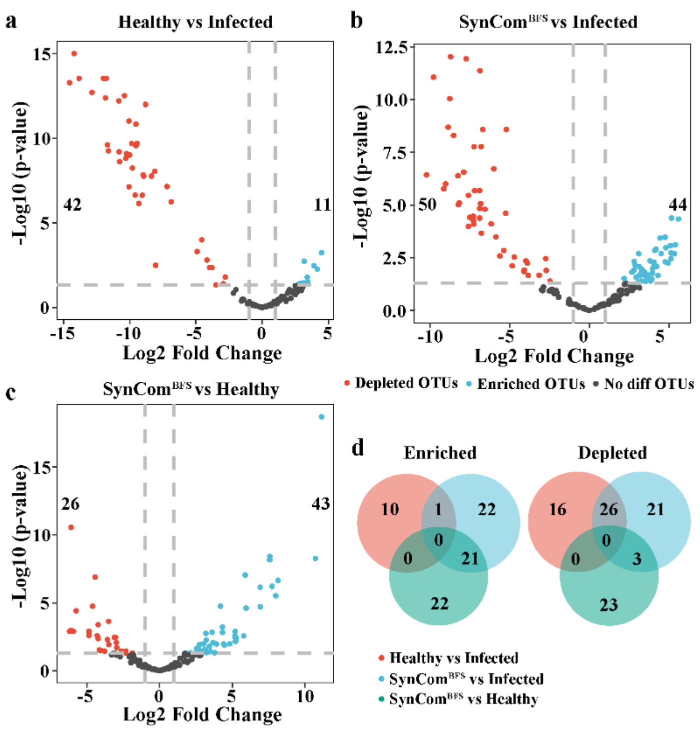

Figure S15 Effects of SynCom^{BFS} addition on the gut bacterial community compositions of shrimp after 56 d of culture. (a) Volcano plot of discriminatory OTUs between healthy and infected shrimp in CK group. (b) Volcano plot of discriminatory OTUs between infected shrimp in CK group and shrimp in SynCom^{BFS} addition group. (c) Volcano plot of discriminatory OTUs between healthy shrimp in CK group and shrimp in SynCom^{BFS} addition group. (d) Venn analysis of the numbers of significantly discriminatory OTUs ($P_{FDR} < 0.05$) shared by healthy and infected shrimp in CK and SynCom^{BFS} addition groups.
